# Immunosuppressive therapy management during sepsis in kidney transplant recipients: a prospective multicenter study

**DOI:** 10.1186/s13613-025-01523-2

**Published:** 2025-08-10

**Authors:** Valentin Rivet, Adrien Joseph, Romain Arrestier, Laure Calvet, Anne-Sophie Moreau, Côme Bureau, Laurent Argaud, Paul Gabarre, Benjamin Zuber, Jean-Herlé Raphalen, Stéphanie Pons, Raphaël Clere-Jehl, Lara Zafrani

**Affiliations:** 1 Medical Intensive Care Unit, Saint-Louis Hospital, Assistance Publique-Hôpitaux de Paris, Paris, France; 2 Medical and Surgical Intensive Care Unit, University Hospital Ambroise Paré, GHU Paris-Saclay, Assistance Publique Hôpitaux de Paris, Boulogne-Billancourt, France; 3 Medical Intensive Care Unit, Henri Mondor Hospital, Assistance Publique-Hôpitaux de Paris, Créteil, France; 4 Medical Intensive Care Unit, Clermont-Ferrand University Hospital, Clermont-Ferrand, France; 5 Medical Intensive Care Unit, Lille University Salengro Hospital, Lille, France; 6 Medical Intensive Care Unit (R3S Department), Pitié-Salpêtrière Hospital, Assistance Publique-Hôpitaux de Paris, Paris, France; 7 Medical Intensive Care Unit, Edouard Herriot Hospital, Hospices Civils de Lyon, Lyon, France; 8 Medical Intensive Care Unit, Saint Antoine Hospital, Assistance Publique-Hôpitaux de Paris, Paris, France; 9 Medical Intensive Care Unit, Hospital Foch, Suresnes, France; 10 Medical Intensive Care Unit, Necker-Enfants Malades Hospital, Assistance Publique-Hôpitaux de Paris, Paris, France; 11 GRC 29, AP-HP, DMU DREAM, Department of Anaesthesiology and Critical Care, Pitié-Salpêtrière Hospital, Sorbonne University, Paris, France; 12 Pulmonary and Critical Care Medicine Division, Department of Medicine, Brigham and Women’s Hospital, Harvard Medical School, Boston, MA 02115 USA; 13 Medical Intensive Care Unit, Strasbourg University Nouvel Hôpital Civil, Strasbourg, France; 14 INSERM UMR 1342, Université Paris Cité, Paris, France

**Keywords:** Kidney transplant, Sepsis, Intensive Care Unit, Immunosuppressive therapy

## Abstract

**Background:**

Sepsis is the leading cause of Intensive Care Unit (ICU) admissions in kidney transplant recipients (KTRs). However, the optimal immunosuppressive therapy (IST) management in this context is not well-defined. We aimed to evaluate the impact of IST management in the ICU on mortality rates and kidney graft function 6 months after inclusion in KTRs admitted for sepsis.

**Methods:**

We conducted a multicenter, prospective, observational study over 1 year in 11 French ICUs. Inclusion criteria were all KTRs who have been transplanted for at least 3 months, admitted to the ICU for sepsis. All changes of IST (7 days prior to ICU admission or throughout the ICU stay) were collected. The primary outcome was MAKE 180 (Major Adverse Kidney Event), a composite outcome including mortality, kidney graft dysfunction and dialysis requirement at 180 days after inclusion.

**Results:**

One hundred and twenty-four patients were included. The main cause of ICU admission was respiratory failure for 78 patients (62.9%). Predominant IST management was mycophenolic acid (MPA) discontinuation for 74 patients (59.7%) and calcineurin inhibitor (CNI) continuation for 63 patients (50.8%). By multivariable analysis, after adjustment for age, non-renal SOFA score at admission, kidney function at admission, sex, and history of cellular rejection we did not find any significant association between MAKE 180 and CNI discontinuation (adjusted OR = 1.05, 95% CI 0.87–1.26, p = 0.6). In contrast, MPA discontinuation was significantly associated with MAKE 180 (adjusted OR = 1.45, 95% CI 1.07–1.96, p = 0.018). No significant association was found between IST discontinuation and ICU-acquired infections (adjusted OR = 1.14, 95% CI 0.95–1.36, p = 0.157). Among ICU survivors, only 2 graft rejections occurred during the year following ICU discharge.

**Conclusion:**

This study is the first prospective investigation to suggest an association between MPA discontinuation and adverse outcomes during sepsis in critically-ill KTRs. These findings must be interpreted with caution given the potential confounding introduced by SARS-Cov-2–specific treatment protocols. Further interventional trials are necessary to optimize immunosuppressive drug strategies in KTRs during sepsis.

**Supplementary Information:**

The online version contains supplementary material available at 10.1186/s13613-025-01523-2.

## Background

Kidney transplantation emerges as the primary therapeutic avenue for individuals with end-stage renal disease. It boasts superior cost-effectiveness and reduced mortality rates compared to long-term dialysis, ultimately enhancing patients' quality of life [1–6]. However, kidney transplantation recipients (KTR) may experience life-threatening complications necessitating Intensive Care Unit (ICU) admission, including severe infections, drug-related toxicities, and cardiovascular events [7].

KTRs exhibit immunosuppression resulting from immunosuppressive therapy (IST) aimed at preventing graft rejection. Standard IST typically includes a calcineurin inhibitor (CNI) combined with an antimetabolite, such as mycophenolic acid (MPA) or azathioprine (AZA), or an mTOR pathway inhibitor (mTORi), along with corticosteroids. In some cases, CNIs can be replaced by belatacept. CNIs suppress T lymphocyte activation by inhibiting the calcineurin pathway, thereby preventing cytokine production and subsequent T cell activation, proliferation, and differentiation [8, 9]. Belatacept, a fusion protein comprising a constant immunoglobulin fragment and CTLA-4 protein, blocks the costimulatory signals required for T lymphocyte activation [10, 11]. Antimetabolites inhibit lymphocyte proliferation by interfering with DNA and RNA synthesis [12, 13]. mTORi impair T lymphocyte proliferation, survival, and protein synthesis [14, 15]. Corticosteroids modulate lymphocyte activity through mechanisms such as suppression of pro-inflammatory cytokine transcription, inhibition of leukocyte migration, and induction of lymphocyte apoptosis [16].

Infections represent the second leading cause of mortality among KTRs, following cardiovascular diseases, and contribute to 15–21% of deaths [17, 18]. Furthermore, infections are correlated with a decline in transplant survival [19] and stand as the primary cause for ICU admission among KTRs, increasing the risks of mortality and graft loss [7].

A remaining question concerns the potential benefits of reducing or discontinuing part or all the IST during sepsis to bolster the immune system's response. The effectiveness of this approach remains unconfirmed. While the anticipated benefit is a reduction in KTR mortality, there is a concomitant risk of inducing an allo-immunization against the kidney graft.

To date, consensus remains elusive regarding the optimal management of IST during sepsis in KTRs. Broadly, the recommendation leans towards a reduction of IST in KTRs with sepsis. Similarly, during the COVID-19 pandemic, the ERA-EDTA and the DESCARTES working group published an expert opinion about the management of immunosuppressive drugs for COVID-19 infected KTRs [20]. For a severe COVID-19 infection, discontinuation of all immunosuppressive drugs (or continuing low-dose CNI in case of a higher immunological risk) and increase of the steroids was advised. However, these recommendations rely on scarce data and results stemming from retrospective data are conflicting on this matter. While some studies indicated that IST discontinuation in solid organ transplant recipients was not associated with acute rejection [21, 22], other studies suggested that IST discontinuation was correlated with poorer outcomes in ICU [23–25]. Therefore, practices remain highly heterogeneous, varying among countries and centers, as demonstrated by Shepshelovich et al. in a multicentric survey-study involving 381 high-volume kidney and liver transplant centers in the US and Europe, which assessed the management of IST during bacterial infections in solid organ transplant recipients [26].

Hence, it is imperative to ascertain whether discontinuing or reducing the dosage of immunosuppressive drugs for KTRs hospitalized in the ICU due to sepsis yields beneficial outcomes. To address this crucial question, we initiated a multicentric prospective observational study assessing the management of IST and its impact on mortality, graft function, and graft rejection. The present study involves a comprehensive follow-up period of 1-year post-inclusion for KTRs admitted to the ICU due to sepsis.

## Patients and methods

### Study design and data collection

In this prospective multicentric observational study, data were collected from 11 French ICUs, using anonymized forms completed by the investigators from each corresponding center. The included patients were KTRs admitted to one of the participating ICUs from November 2021 to November 2022, due to sepsis, according to the Sepsis-3 campaign definition [27]. Excluded from the study were patients under 18 years old, pregnant women, KTRs transplanted within the last 3 months prior to ICU admission, KTRs with heart and/or lung transplant. We prospectively collected the following data on each patient: demographic data, history of kidney transplantation, history of opportunistic infections, history of cancer, CMV serological status, cardiovascular comorbidities, infectious prophylaxis, chronological characteristics of hospitalization (date of admission to hospital and ICU, date of modification of IST, date of discharge from ICU), initial IST upon hospitalization and/or ICU admission, and any modifications to this treatment, SOFA (Sepsis-related Organ Failure Assessment) score within the first 24 h after ICU admission, as previously defined [28], type of infection (pathogen involved, location of infection), kidney graft function at baseline, on ICU discharge, at 6 months and 1 year post-inclusion, occurrence of acute kidney injury (AKI), need for renal replacement therapy (RRT) and the chosen technique (intermittent hemodialysis, continuous veno-venous hemofiltration, continuous veno-venous hemodiafiltration), need and duration of catecholamine support and mechanical ventilation, and in-hospital mortality. We collected data at 6 months and 1 year after ICU admission: occurrence of death, occurrence of antibody mediated rejection (AMR) and/or T-cell mediated rejection (TCMR), Donor Specific Antibodies (DSA), and graft function or need for RRT.

### Definitions

IST management was classified as a discontinuation if an immunosuppressive drug was stopped for at least 24H, or continuation if the drug was continued even with a dose reduction. This change had to occur in the ICU or less than 7 days before ICU admission. Graft function was assessed by serum creatinine and estimated Glomerular Filtration Rate (eGFR) according to CKD-EPI [29]. AKI was assessed and staged according to KDIGO criteria [30]. Baseline serum creatinine was defined as the serum creatinine measured in the 3 months preceding the hospitalization in the ICU. Patients severity at ICU admission was assessed using the SOFA score (Sepsis-related Organ Failure Assessment) [28], and extra-renal SOFA was calculated as the SOFA score without considering kidney failure. CNI overdose was defined as a residual tacrolimus (T0) level > 10 ng/mL or a residual cyclosporine (C0) level > 250 ng/mL. AMR and TCMR were diagnosed by the nephrologist team caring for the KTR, according to the Banff Classification after a kidney graft biopsy [31]. ICU-acquired infection was defined by any infection occurring more than 48 h after ICU admission, as previously defined [32].

### Endpoints

To assess the impact of IST management, we used as primary endpoint a composite outcome of death, new RRT, and worsened graft function, defined as a 25% or greater decline in eGFR, constituting the major adverse kidney event (MAKE) outcome [33]. MAKE outcomes have been previously assessed and validated in different ICU studies until 1 year after discharge [34–36]. We assessed this composite outcome (death, use of RRT and worsened graft function) at 6 months (180 days) after inclusion (MAKE 180). As secondary endpoints, we assessed MAKE at ICU discharge and MAKE 360 (360 days after inclusion), and the impact of IST management on ICU-acquired infections. Given the non-interventional design of our study, assessments could not be performed exactly at 180 or 360 days post-inclusion for all patients. Therefore, we defined acceptable time windows of ± 1 month for MAKE 180 and ± 2 months for MAKE 360.

### Statistical analysis

Continuous data were described as median (interquartile range (IQR)). Categorical data were presented as numbers and percentages of total. Comparisons were made using a Wilcoxon rank sum test (continuous variables) or Fisher exact test (categorical variables). Variables significantly associated with outcomes in univariate analysis and clinically significant were included in multivariable logistic regression models with backward stepwise selection and odds ratio (OR) were calculated. Cumulative incidence plots were stratified according to IST management and compared using a log-rank test. Temporal relationship between IST management and AKI was depicted with density curves. P values less than 0.05 were considered statistically significant, and all statistical tests were 2-sided. Statistical analyses were performed using R statistical software [R Core Team (2023). _R: A Language and Environment for Statistical Computing_. R Foundation for Statistical Computing, Vienna, Austria. (https://www.R-project.org/)].

## Results

### Patients’ characteristics

**Fig. 1:**
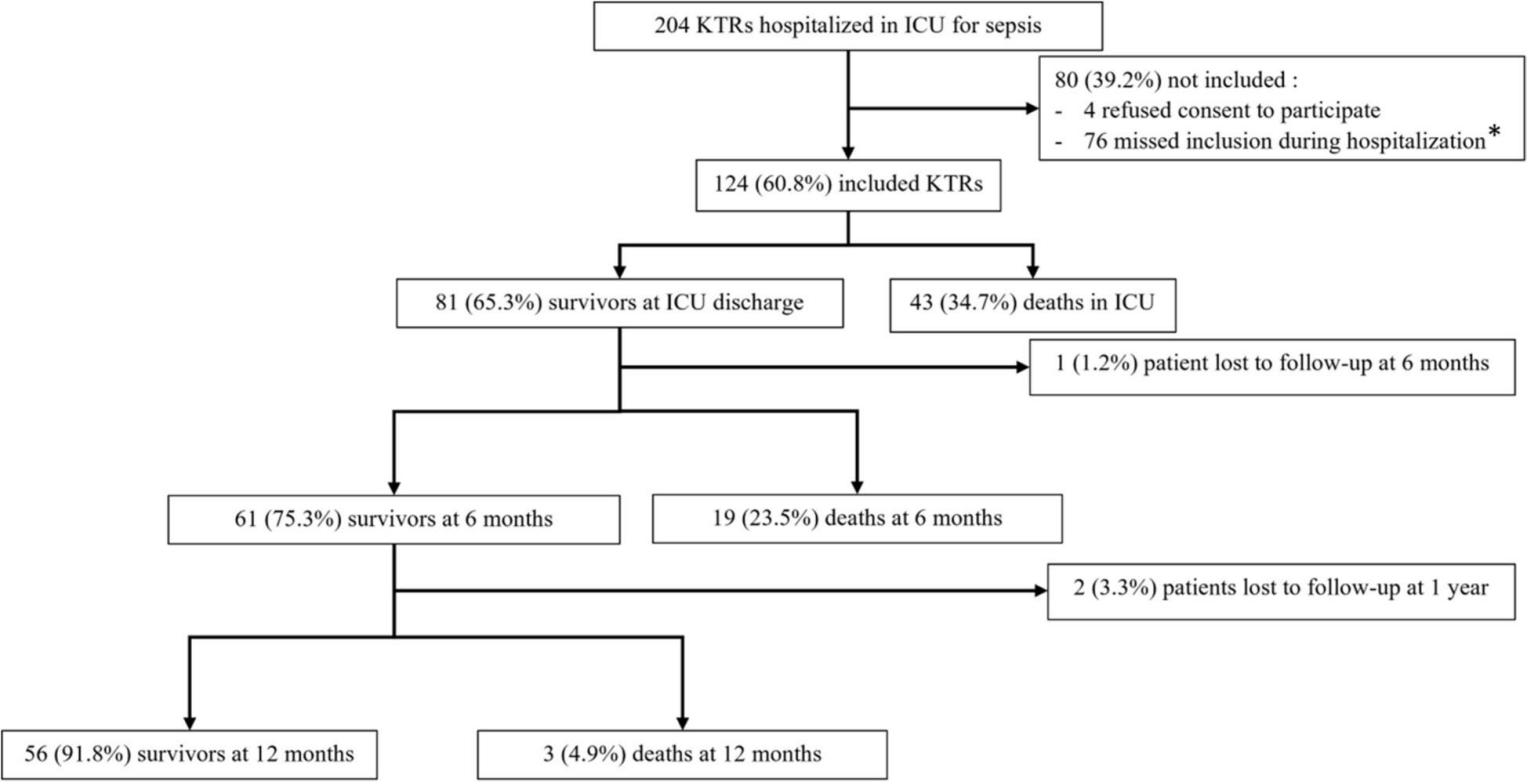
Flowchart. KTR: Kidney transplant recipients; ICU: Intensive care unit. *These 76 patients were not included due to non-systematic screening, limited investigator availability, or logistical limitations

Two hundred and four KTRs were admitted for sepsis in one of the 11 participating centers between November 2021 and November 2022 (Fig. 1). Among them, 124 (60.8%) were finally included in the study. Eighty KTRs were not enrolled: 4 declined to provide consent, and 76 were not included due to investigator oversight or failure to include at the time of ICU admission. Patient characteristics are reported in Table 1.

**Table 1:** Patients characteristics is it possible to improve the readibility of the Tables 1, Table 2 and Table 5 to make the subdivisions more visually distinct (maybe clearer spacing between subcategories) as in the originally submitted Tables ? Many thanks

	Total (n = 124)	MAKE 180 = 0 (n = 38)	MAKE 180 = 1 (n = 85)	p
Median age (IQR), years	54 (54.8–70)	60 (53.3–65.8)	65 (57–71)	0.1
Gender, male, n (%)	74 (59.7)	31 (81.6)	42 (49.4)	0.002
ESRD etiology, n (%)				0.4
Diabetes mellitus	35 (28.2)	9 (23.7)	26 (30.6)	
Hypertension	11 (8.9)	1 (2.6)	9 (10.6)	
CGN	23 (18.5)	5 (13.2)	18 (21.2)	
ADPKD	8 (6.5)	4 (10.5)	4 (4.7)	
Others	47 (37.9)	19 (50)	28 (32.9)	
Modality of pre-transplant dialysis, n (%)				
Hemodialysis	124 (100)	38 (100)	85 (100)	1
Peritoneal dialysis	0 (0)	0 (0)	0 (0)	1
Comorbidities, n (%)				
Malignancy	24 (19.4)	5 (13.2)	19 (22.4)	0.326
Other cause of immunosuppression	14 (11.3)	3 (7.9)	11 (12.9)	0.546
Coronary artery disease	29 (23.4)	10 (26.3)	19 (22.4)	0.651
Cerebrovascular disease	6 (4.8)	1 (2.6)	5 (5.9)	0.665
Peripheral artery disease	18 (14.5)	3 (7.9)	15 (17.7)	0.181
Diabetes mellitus	66 (53.2)	17 (44.7)	49 (57.7)	0.258
Hypertension	108 (87.1)	34 (89.5)	74 (87.7)	0.999
History of allograft rejection, n (%)				
TCMR	17 (13.7)	10 (26.3)	7 (8.2)	0.011
AMR	23 (18.5)	9 (23.7)	14 (16.5)	0.45
History of DSA (n = 62)				0.17
DSA	25 (20.2)	14 (51.9)	11 (31.4)	
No DSA	37 (29.8)	13 (48.1)	24 (68.6)	
History of opportunistic infection, n (%)	55 (44.4)	13 (34.2)	42 (49.4)	0.171
Viral OI	25 (20.2)	3 (7.9)	22 (25.9)	0.041
Bacterial OI	3 (2.4)	1 (2.6)	2 (2.4)	1
Fungal OI	11 (8.9)	2 (5.3)	9 (10.6)	0.54
Median time between transplantation and ICU admission (IQR), months	72 (27–126.5)	74 (28.5–157)	71.5 (27–121)	0.648
Median creatinine at baseline (IQR), µmol/L	149 (110–200)	159 (111.7–211.5)	147 (107–200)	0.35
Median eGFR at baseline (IQR), ml/min/1.73 m^2^	39 (28.5–58)	37.3 (29.3–57.8)	39 (27.6–56.9)	0.88
Baseline immunosuppressive regimen, n (%)				
CNI	103 (83.1)	30 (78.9)	72 (84.7)	0.45
MPA	90 (72.6)	31 (81.6)	59 (69.4)	0.24
AZA	8 (6.5)	2 (5.3)	6 (7.1)	1
mTORi	11 (8.9)	2 (5.3)	9 (10.6)	0.5
Corticosteroids	109 (87.9)	34 (89.5)	74 (87.1)	0.78
Belatacept	12 (9.7)	4 (10.5)	8 (9.4)	1
Number of immunosuppressive drugs at ICU admission, n (%)				0.91
4	1 (0.8)	0 (0)	1 (1.2)	
3	85 (68.5)	28 (73.7)	57 (67.1)	
2	36 (29)	9 (23.7)	26 (30.6)	
1	2 (1.6)	1 (2.6)	1 (1.2)	
Median duration of ICU stay (IQR), days	7 (3–15.5)	4 (3–9.5)	8.4 (4–15)	0.017
Median SOFA score at ICU admission (IQR)	5 (4–7)	4 (3–5)	6 (5–8)	< 0.001
Cause of ICU admission, n (%)				0.83
Hemodynamic failure	40 (32.3)	13 (34.2)	27 (31.8)	
Respiratory failure	78 (62.9)	23 (60.5)	55 (64.7)	
Neurological failure	6 (4.8)	2 (5.3)	3 (3.5)	
IST at ICU admission, n (%)				
CNI	101 (81.5)	30 (78.9)	70 (82.4)	0.61
MPA	67 (54)	24 (63.2)	43 (50.6)	0.27
AZA	6 (4.8)	2 (5.3)	4 (4.7)	1
mTORi	12 (9.7)	2 (5.3)	10 (11.8)	0.34
Corticosteroids	110 (88.7)	33 (86.8)	76 (89.4)	0.76
Median IST dosage at ICU admission (IQR)				
Tacrolimus T0, ng/mL (n = 66)	6.5 (4.2–9.95)	5.45 (3.7–11)	6.8 (5–9.9)	0.39
Ciclosporin C0, ng/mL (n = 15)	85 (55.5–132.5)	88 (74.3–125.8)	78 (55.5–132.5)	0.95
MPA, mg per day (n = 62)	1000 (1000–1500)	1500 (1000–1500)	1000 (1000–1500)	0.26
Prednisone, mg per day (n = 81)	7.5 (5–10)	10 (5–10)	7.5 (5–10)	0.78

MAKE 180 = 0: MAKE outcome not achieved at 180 days after inclusion. MAKE 180 = 1: MAKE outcome achieved 180 days after inclusion. ESRD: End Stage Renal Disease. CGN: Chronic Glomerulonephritis. ADPKD: Autosomal Dominant Polycystic Kidney Disease. TCMR: T cell mediated rejection. AMR: Antibody Mediated Rejection. DSA: Donor specific Antibodies. OI: Opportunistic infection. eGFR: estimated Glomerular Filtration Rate. CNI: Calcineurin Inhibitor. MPA: Mycophenolic Acid. AZA: Azathioprine. mTORi: mTOR inhibitor. SOFA: Sepsis-related Organ Failure Assessment

Eighty-five (68.5%) patients were on triple IST. Immunosuppressive drugs are detailed in Table 1. Seventy patients (56.5%) were previously admitted to a conventional hospitalization unit before ICU admission, during which their IST could have been modified. Within the first 24 h of ICU admission, the median SOFA score was 5 (IQR = 4–7), and the first cause of admission was respiratory failure for 78 patients (62.9%). Among the 124 included patients, 43 (34.7%) died during their ICU stay, 81 (65.3%) survived. Among survivors, 22 (27.2%) died within 12 months of their hospitalization.

Eighty-five patients (69.1%) met the MAKE 180 criteria, while 38 patients (30.9%) did not. One patient was lost to follow-up. Among the 85 patients who met the MAKE 180 criteria, 62 (72.9%) had died at 6 months (including 43 who died during their ICU stay and 19 who died in the 6 months following ICU discharge), 7 patients (8.2%) required renal replacement therapy (RRT), and 16 patients (18.8%) experienced graft dysfunction at 6 months (Table S1).

Fifty-four patients (43.5%) met the MAKE at ICU discharge criteria, and 70 patients (56.5%) did not. Among the 54 patients who met the MAKE at ICU discharge criteria, 43 (79.6%) had died, 2 (3.7%) required RRT, and 9 (16.7%) experienced graft dysfunction at ICU discharge.

Eighty-nine patients (73.6%) met the MAKE 360 criteria, while 32 patients (26.4%) did not. Three patients were lost to follow-up. Among the 89 patients who met the MAKE 360 criteria, 65 (73%) had died, 8 (9%) required RRT, and 16 (18%) experienced graft dysfunction at 1 year. (Table S2).

All deaths in the ICU were due to infectious causes. Sixteen (37.2%) patients died from refractory acute respiratory distress syndrome, 19 (44.2%) from multiorgan failure secondary to refractory septic shock, 8 (18.6%) following the withdrawal of life-sustaining treatments and 1 (2.3%) from brain death. The most frequent causes of ICU admission among non-survivors were respiratory failure (n = 30), followed by hemodynamic failure (n = 12) and neurological failure (n = 1).

Among the 22 patients who died after ICU discharge, 13 (59.1%) died from infectious causes, 4 (18.2%) from neoplastic causes, 1 (4.5%) from hemorrhagic shock, and in 4 (18.2%) cases, cause of death was unavailable.

### ICU management

In this study, any discontinuation of at least one immunosuppressive drug (excluding belatacept, which is administered monthly) for at least 24 h, occurring within 7 days before ICU admission or during the ICU stay, was recorded. Thus, 100 patients (80.6%) experienced a discontinuation of at least one immunosuppressive drug. Among the 103 patients on CNI, 40 (38.8%) had a discontinuation compared to 63 (61.2%) who continued their treatment, representing 32.3% and 50.8% of the total population respectively (Table 2). Among them, 2 patients had a CNI discontinuation occurring one day before ICU admission and the second one occurring the day of ICU admission. The median time to CNI suspension was within 24 h following ICU admission (IQR = 0–1) (Figure S1). The primary reasons for CNI discontinuation in the ICU were AKI related to nephrotoxicity and/or overdose. Among the 40 patients who experienced CNI discontinuation, residual tacrolimus (T0) or cyclosporine (C0) levels were available for 30 patients (75%). Among these 30 patients, 10 (33.3%) exhibited CNI overdose, and all 10 presented with AKI upon ICU admission. Furthermore, of the 40 patients with CNI discontinuation, 36 (90%) had AKI at ICU admission, while the remaining 4 patients (10%) did not. We did not identify any CNI toxicity attributable to ICU administered drug interactions in our cohort. No discontinuation appeared to be due to gut failure.

**Table 2:** Outcomes according to IST management in ICU

	Total (n = 124)	ICU mortality (n = 43)	MAKE at ICU discharge = 1 (n = 54)	MAKE 180 = 1 (n = 85)	MAKE 360 = 1 (n = 89)	1 year post-ICU rejection (n = 2)	Persistent graft dysfunction or need for hemodialysis at 1 year post-ICU (n = 25)
Immunosuppressive regimen management, n (%)							
CNI discontinuation	40 (32.3)	14 (32.6)	20 (37)	33 (38.8)	32 (36)	1 (50)	11 (44)
CNI continuation	63 (50.8)	22 (51.2)	26 (48.1)	39 (45.9)	43 (48.3)	1 (50)	10 (40)
CNI dose reduction	10 (8.1)	4 (9.3)	4 (7.4)	6 (7.1)	6 (6.7)	0 (0)	0 (0)
No change of CNI dose	53 (42.7)	18 (41.9)	22 (40.7)	33 (38.8)	37 (41.6)	1 (50)	10 (40)
MPA discontinuation	74 (59.7)	29 (67.4)	35 (64.8)	52 (61.2)	51 (57.3)	1 (50)	11 (44)
MPA continuation	10 (8.1)	0 (0)	0 (0)	2 (2.4)	5 (5.6)	0 (0)	4 (16)
MPA dose reduction	1 (0.8)	0 (0)	0 (0)	0 (0)	0 (0)	0 (0)	0 (0)
No change of MPA dose	9 (7.3)	0 (0)	0 (0)	2 (2.4)	5 (5.6)	0 (0)	4 (16)
AZA discontinuation	7 (5.6)	1 (2.3)	1 (1.9)	5 (5.9)	5 (5.6)	0 (0)	2 (8)
AZA continuation	0 (0)	0 (0)	0 (0)	0 (0)	0 (0)	0 (0)	0 (0)
AZA dose reduction	0 (0)	0 (0)	0 (0)	0 (0)	0 (0)	0 (0)	0 (0)
No change of AZA dose	0 (0)	0 (0)	0 (0)	0 (0)	0 (0)	0 (0)	0 (0)
mTORi discontinuation	9 (7.3)	4 (9.3)	4 (7.4)	9 (10.6)	8 (9)	0 (0)	2 (8)
mTORi continuation	3 (2.4)	0 (0)	0 (0)	1 (1.2)	1 (1.1)	0 (0)	0 (0)
mTORi dose reduction	0 (0)	0 (0)	0 (0)	0 (0)	0 (0)	0 (0)	0 (0)
No change of mTORi dose	3 (2.4)	0 (0)	0 (0)	1 (1.2)	1 (1.1)	0 (0)	0 (0)
Corticosteroid discontinuation	2 (1.6)	2 (4.7)	2 (3.7)	2 (2.4)	2 (2.2)	0 (0)	0 (0)
Corticosteroid continuation	111 (89.5)	39 (91)	49 (90.7)	76 (89.4)	81 (91)	2 (100)	22 (88)
Corticosteroid dose reduction	2 (1.6)	1 (2.3)	1 (1.9)	1 (1.2)	1 (1.1)	0 (0)	0 (0)
No change of corticosteroid dose	109 (87.9)	38 (88.4)	48 (88.9)	75 (88.2)	80 (89.9)	2 (100)	22 (88)
Organ support							
Vasopressors	57 (46)	33 (76.7)	40 (74.1)	49 (57.7)	49 (55.1)	2 (100)	8 (33.3)
Mechanical ventilation	52 (41.9)	35 (81.4)	40 (74.1)	46 (54.1)	45 (50.6)	1 (50)	6 (25)
Renal replacement therapy	27 (21.8)	18 (41.9)	23 (42.6)	26 (30.6)	26 (29.2)	0 (0)	3 (12.5)

MAKE at ICU discharge = 1: MAKE outcome achieved at discharge. MAKE 180 = 1: MAKE outcome achieved 180 days after inclusion. MAKE 360 = 1: MAKE outcome achieved 360 days after inclusion. CNI: Calcineurin inhibitor. MPA: Mycophenolic acid. AZA: Azathioprine. mTORi: mTOR inhibitor

Eighteen patients (45% of those with CNI discontinuation) had CNI reinitiated in the ICU, with a median time of 4.5 days after suspension (IQR = 2.25–5.75).

Among the 90 (72.6%) patients initially on mycophenolic acid (MPA), 74 (82.2% of patients on MPA) experienced a discontinuation of treatment compared to 10 (11.1% of patients on MPA) who did not, representing 59.7% and 8.1% of the total population, respectively. Clinicians discontinued MPA due to neutropenia in 2 patients (2.7% of patients with MPA discontinuation) or to reduce immunosuppression in 72 patients (97.3% of patients with MPA discontinuation). Six patients (6.7% of patients on MPA) had MPA discontinuation more than 7 days before ICU admission, and seventeen had MPA discontinuation within 7 days before ICU admission, with a median time of 2 days between discontinuation and ICU admission (IQR = 1–3.75) (Figure S2). For the remaining 57 patients, MPA was predominantly suspended within 24 h of ICU admission. Only 2 patients had their corticosteroid therapy suspended, and 22 patients had a switch for hydrocortisone hemisuccinate. No patient on belatacept received a new injection of the treatment in the ICU. Therefore, the predominant management of immunosuppression was MPA discontinuation for 59.7% of patients and CNI continuation for 50.8% of patients (Table 2).

Among patients who had MPA discontinuation, a larger proportion died in the ICU (39.2% vs. 0%, p = 0.013) and met the MAKE at ICU discharge (47.3% vs. 0%, p < 0.001) and MAKE 180 criteria (70.3% vs. 20%, p < 0.001), in comparison to MPA continuation. But MPA management did not significantly differ for MAKE 360 (68.9% vs. 50%, p = 0.16) and the persistence of graft dysfunction or dialysis at 1 year (14.9% vs. 40%, p = 0.07).

We analyzed the association between clinical severity and IST management. A higher median SOFA score at admission was associated with discontinuation of at least one IST including CNI and/or MPA discontinuation (Table 3). A higher median AKI grade according to KDIGO classification was also associated with CNI discontinuation. Similarly, discontinuation of at least one IST or MPA was more frequent when patients required mechanical ventilation, and discontinuation of CNI was more frequent when patients required vasopressors (Table 3). Lastly, the median duration of ICU stay was longer in patients experiencing MPA discontinuation than MPA continuation (Table 3).

**Table 3:** IST management according to patients’ severity and immunological risk

	IST discontinuation (n = 100)	IST continuation (n = 24)	p	CNI discontinuation (n = 40)	CNI continuation (n = 63)	p	MPA discontinuation (n = 74)	MPA continuation (n = 10)	p
Median SOFA at admission (IQR)	5.5 (4–7)	4 (4–5)	0.007	7 (5–10)	5 (3–6)	0.001	5 (4–7)	4 (3.25–4.75)	0.033
Median KDIGO grade of AKI (IQR), (n = 103)	2 (1–3)	2 (1–2)	0.075	2 (2–3)	2 (1–2)	0.001	2 (1–3)	1 (1–2)	0.098
RRT, n (%), (n = 27)	24 (24)	3 (13)	0.279	11 (28)	9 (14)	0.127	21 (28)	0 (0)	0.06
Use of vasopressors, n (%), (n = 57)	50 (50)	7 (29)	0,107	25 (63)	24 (38)	0,027	35 (47)	1 (10)	0,058
Median duration of vasopressor use (IQR), days, (n = 56)	4 (2–8)	3 (1.5–5.25)	0.191	3 (2–5)	5 (2–8)	0.739	5 (2–8)	1 (1–1)	NA
Median maximal norepinephrine dosage (IQR), mg/h, (n = 33)	2.2 (1.5–5)	0.7 (0.6–1)	0.02	2.5 (2–6.5)	1.75 (0.8–2.4)	0.056	2 (1.4–3.2)	0.7 (0.7–0.7)	NA
Median lactate peak (IQR), mmol/L (n = 105)	2.1 (1.3–3.5)	1.7 (1.3–2.7)	0.236	2.3 (2–3.6)	1.8 (1.2–3.2)	0.064	2.1 (1.45–3.5)	1.3 (1.23–2.93)	0.682
Median number of days between ICU admission and lactate peak (IQR), days, (n = 104)	1 (0–5.25)	2 (0–5)	0.779	1 (1–4)	1 (0–5)	0.561	2 (0–6)	0 (0–0.75)	0.052
Mechanical ventilation, n (%), (n = 52)	47 (47)	5 (21)	0.036	21 (53)	23 (37)	0.163	40 (54)	0 (0)	0.001
Median duration of mechanical ventilation (IQR), days	8 (4–17.75)	14.5 (9.5–21)	0.237	7.5 (3.75–22.5)	9.5 (4.25–13.75)	0.96	8 (4–19)	0 (0–0)	NA
Median duration of ICU stay (IQR), days	7 (4–15)	5 (3–11)	0.238	6 (4–14.25)	5.5 (3–12.75)	0.296	9 (4–15)	3 (2–4)	0.002
Known DSA, n (%), (n = 63)	20 (20)	5 (21)	0.764	10 (25)	11 (17)	0.253	14 (19)	4 (40)	0.697
History of transplant rejection, n (%)	26 (26)	7 (29)	0.799	9 (23)	15 (24)	0.999	16 (22)	6 (60)	0.018
Number of kidney transplantations, n (%)			0.458			0.708			0.999
1	89 (89)	23 (95.8)		36 (90)	59 (93.7)		64 (86.5)	9 (90)	
2	11 (11)	1 (4.2)		4 (10)	4 (6.3)		10 (13.5)	1 (10)	

IST: Immunosuppressive Therapy. CNI: Calcineurin Inhibitor. MPA: Mycophenolic Acid. SOFA: Sepsis-related Organ Failure Assessment. AKI: Acute Kidney Injury. RRT: Renal Replacement Therapy. DSA: Donor Specific Antibody

We also assessed IST management based on immunological risk factors, including donor-specific antibodies (DSA), prior episodes of rejection, and the number of transplantations. Among these only a history of rejection was associated with more frequent continuation of MPA (Table 3).

Among all patients surviving ICU, only 2 graft rejections occurred after ICU discharge, 1 TCMR and 1 AMR. The first patient received IST consisting of a triple regimen of CNI, MPA, and oral corticosteroids, and had MPA discontinued the day of ICU admission, and during the whole ICU stay. This patient had no history of rejection or DSAs before admission. TCMR was diagnosed 4 weeks after ICU discharge, and the eGFR remained stable at 19 ml/min/1,73m^2^ 1 year after ICU discharge. The second patient, who experienced an AMR, was initially on belatacept, CNI, and oral corticosteroids and had low-level DSAs (maximum Mean Fluorescence Intensity of 500) before ICU admission. Management involved CNI discontinuation 48 h after ICU admission, with no resumption during the 33-day stay. AMR was diagnosed 5 weeks after ICU discharge, but no significant change in eGFR was observed at 6 months and 1 year post-ICU discharge. Standard management of TCMR included three boluses of methylprednisolone. For AMR, treatment comprised methylprednisolone boluses, plasma exchange, and intravenous immunoglobulins. Histological criteria for rejection diagnosis adhered to the Banff classification [31].

**Fig. 2:**
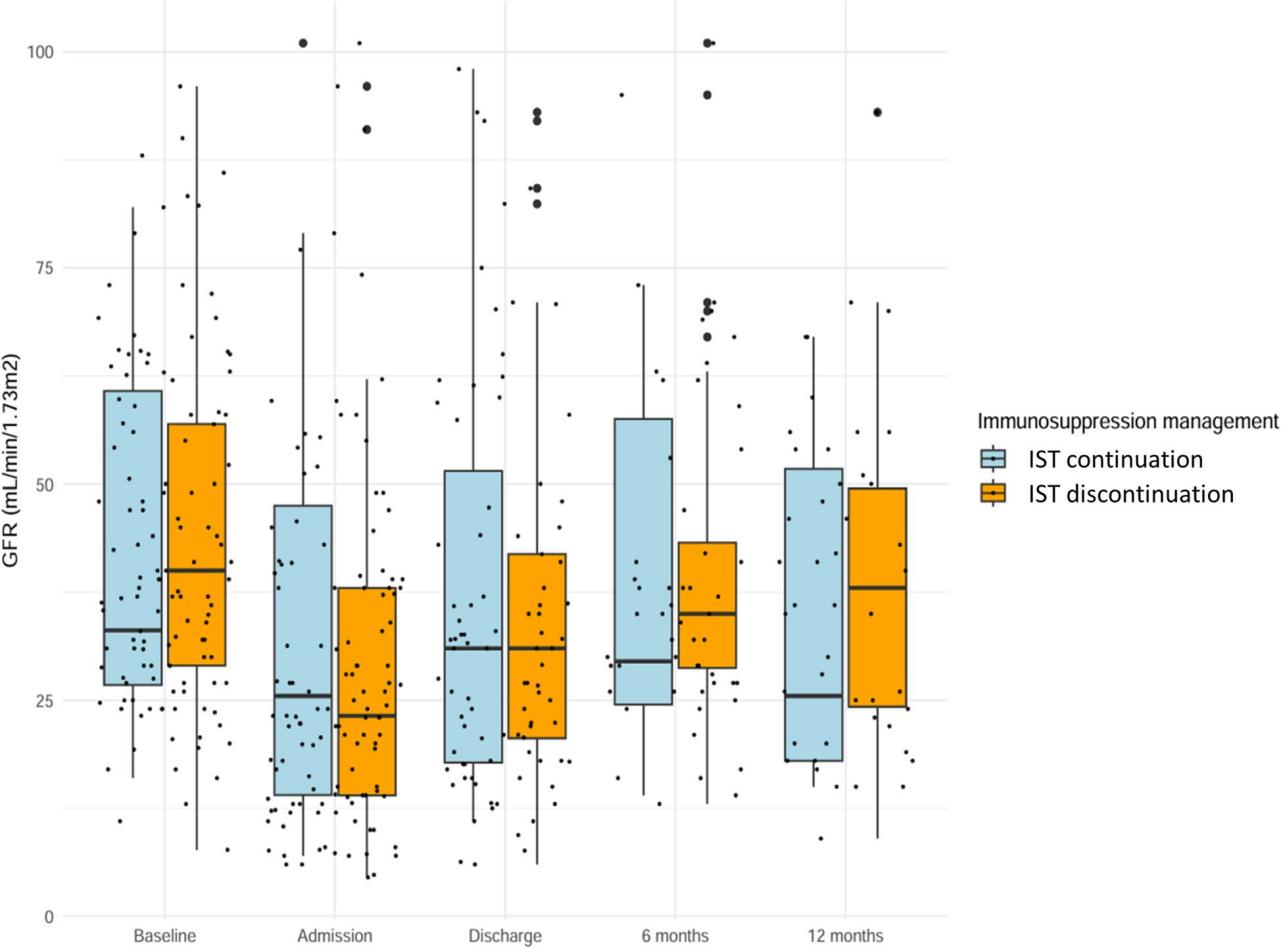
Box plot of patients’ kidney graft function according to immunosuppressive therapy (IST) management in ICU. Each point represents the value of the estimated GFR (glomerular filtration rate) of patients at baseline, ICU admission, ICU discharge, 180 and 360 days after inclusion. Blue box plots represent patients having IST continuation in ICU and yellow box plots represent patients having IST discontinuation in ICU

For surviving KTRs not requiring RRT after ICU, the mean eGFR at 180 and 360 days did not significantly differ between those who had a discontinuation of at least one immunosuppressant and those who did not (Fig. 2) (mean eGFR at 180 days: 40.8 ml/min/1.73m^2^ vs. 38.3 ml/min/1.73m^2^, p = 0.69- and at 360 days: 39.3 ml/min/1.73m^2^ vs. 35.1 ml/min/1.73m^2^, p = 0.51).

### Factors associated with MAKE 180

**Fig. 3:**
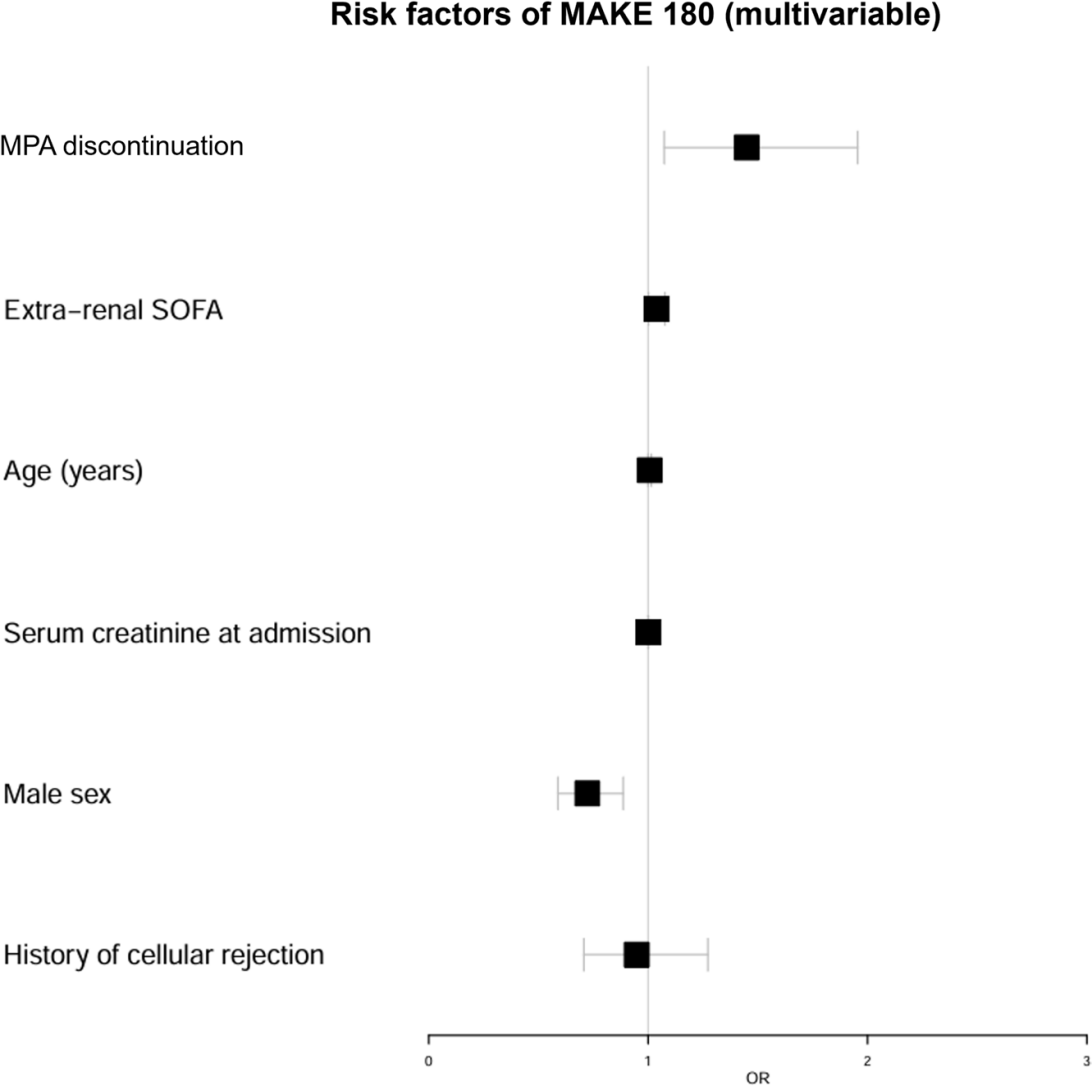
Forest plot of the factors associated with MAKE 180 by multivariable analysis. MPA: Mycophenolic acid; SOFA: Sepsis-related organ failure assessment

**Fig. 4:**
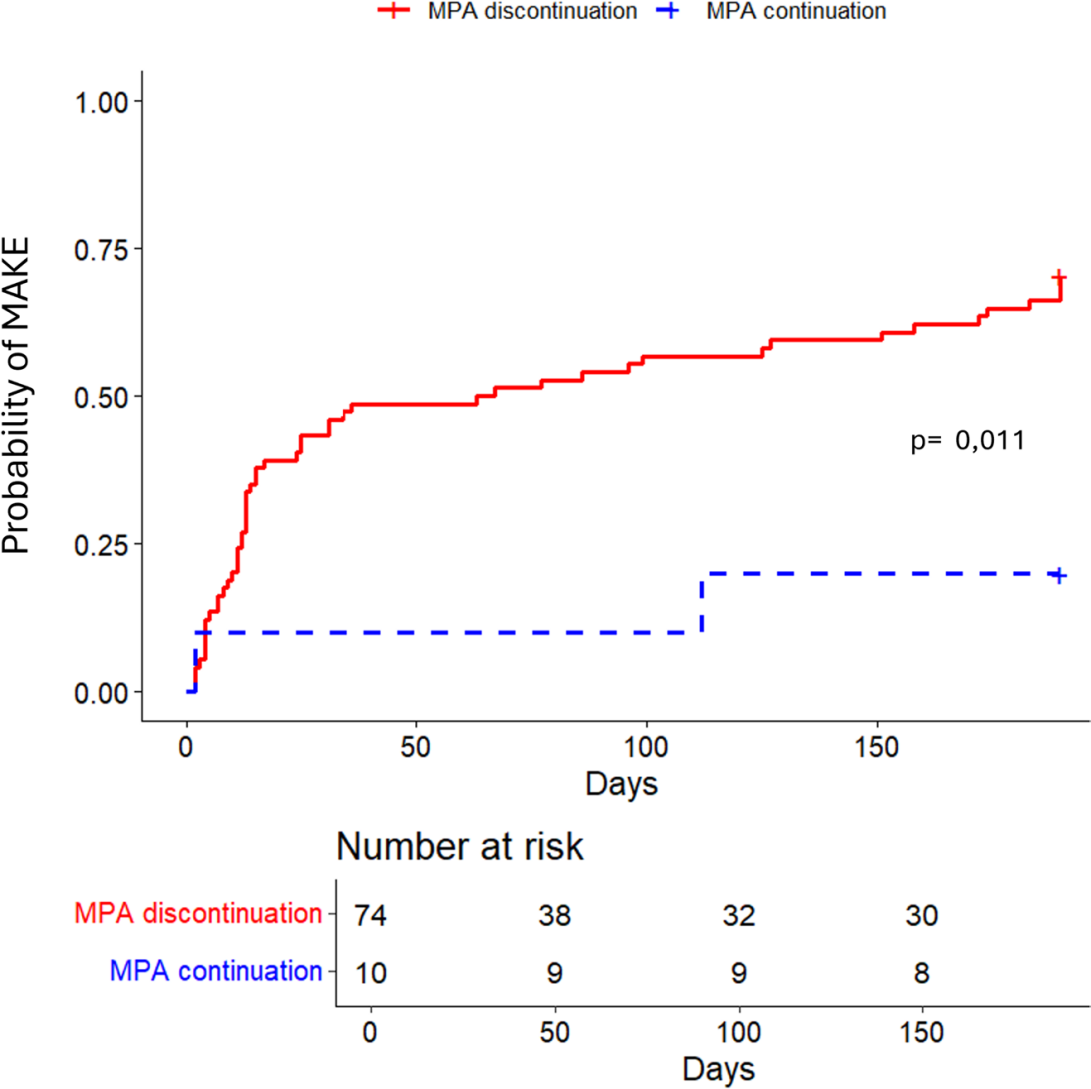
Cumulative incidence plot of MAKE according to MPA management. Patients with MPA continuation in ICU are represented by the blue line and patients with MPA discontinuation in ICU are represented by the red line. MPA: Mycophenolic acid

KTRs who discontinued at least one immunosuppressive drug, CNI, MPA, or mTOR inhibitor (mTORi) were associated to the MAKE 180 criteria in univariate analysis (Table 4). By multivariable analysis, after adjustment for age, non-renal SOFA score at admission, graft function at admission, sex, and history of cellular rejection we did not find any significant association between MAKE 180 and CNI discontinuation (adjusted OR = 1.05, 95% CI 0.87–1.26, p = 0.6), or mTORi discontinuation (adjusted OR = 1.52, 95% CI 0.86–2.67, p = 0.21) (Fig. 3 and Figure S3). In contrast, MPA discontinuation remained significantly associated with the MAKE 180 criteria in multivariable analysis (adjusted OR = 1.45, 95% CI 1.07–1.96, p = 0.018) (Figs. 3 and 4). We conducted an additional analysis to evaluate the impact of different durations of IST discontinuation (48 h and 72 h) on MAKE outcomes. For CNI discontinuation, the association remained non-significant. However, for MPA discontinuation, the association remained significant across all durations analyzed (discontinuation > 24 h: non-adjusted OR = 1.653, 95% CI 1.22–2.234, p = 0.001, discontinuation > 48 h: non-adjusted OR = 1.432, 95% CI 1.093–1.877, p = 0.011, discontinuation > 72 h: non-adjusted OR = 1.447, 95% CI 1.143–1.832, p = 0.003) (Table S3).

**Table 4:** Non-adjusted Odds Ratio of IST management on ICU mortality, AKI, MAKE at ICU discharge and MAKE 180 criteria

	ICU mortality		AKI		MAKE at ICU discharge		MAKE 180	
	OR (95% CI)	p	OR (95% CI)	p	OR (95% CI)	p	OR (95% CI)	p
At least 1 immunosuppressive drug discontinuation	1.187 (0.961–1.467)	0.114	1.164 (0.985–1.374)	0.076	1.195 (0.959–1.490)	0.115	1.371 (1.112–1.68)	0.003
CNI discontinuation	1.01 (0.83–1.231)	0.918	1.193 (1.033–1.378)	0.018	1.089 (0.886–1.337)	0.421	1.21 (1.007–1.453)	0.045
MPA discontinuation	1.480 (1.089–2.01)	0.014	1.148 (0.890–1.480)	0.291	1.605 (1.173–2.195)	0.004	1.653 (1.22–2.234)	0.001
mTORi discontinuation	1.56 (0.842–2.888)	0.188	1.56 (0.842–2.888)	0.188	1.56 (0.842–2.888)	0.188	1.948 (1.39–2.73)	0.003

IST: Immunosuppressive therapy; ICU: Intensive care unit; AKI: acute kidney injury; CNI: Calcineurin inhibitor; MPA: Mycophenolic acid. mTORi: mTOR inhibitor

Among the MAKE criteria components, MPA discontinuation is associated with mortality (non-adjusted OR = 1.480, 95% CI 1.089–2.01, p = 0.014), and not with kidney transplant dysfunction or the need for dialysis, as the occurrence of AKI in the ICU was not associated with MPA discontinuation (non-adjusted OR = 1.15, 95% CI 0.89–1.48, p = 0.29) (Table 4). In contrast, CNI discontinuation was associated with AKI (non-adjusted OR = 1.19, 95% CI 1.03–1.38, p = 0.018), but it was no longer significant after adjustment for kidney function at admission (adjusted OR = 1.15, 95% CI 0.99–1.33, p = 0.07), neither after adjustment for the extra-renal SOFA score (adjusted OR = 1.13, 95% CI 0.97–1.33, p = 0.13), suggesting that CNI discontinuation primarily occurred in the most severe patients. At 180 days, the eGFR decline (relative to baseline eGFR) was significantly greater in patients who had discontinued CNI. This association was not observed for MPA discontinuation, any other IST discontinuation, or at 360 days (Table S4 and Figure S4).

We also analyzed the impact of reducing the number of immunosuppressive drugs during the ICU stay—specifically, from three drugs at baseline to two, from three to one, and from two to one—but no statistically significant association with MAKE 180 was observed (Table S3). Finally, when comparing centers that included fewer than 8 patients to those that included 8 patients or more, center volume was not significantly associated with MAKE 180 (OR = 1.09, 95% CI 0.84–1.43, p = 0.523).

### Infections in ICU

The most common infection at ICU admission was an isolated SARS-CoV-2 pneumonia for 44 (35.5%) KTRs (40 received Dexamethasone 6 mg at admission, and 21 received Tocilizumab), followed by transplant pyelonephritis for 21 (16.9%) patients and bacterial pneumonia for 19 patients (15.3%) (Table 5). Eighteen (14.4%) patients had two concomitant infections at admission, and 23 (18.5%) developed an ICU acquired infection. No significant association was found between IST discontinuation and ICU-acquired infections (adjusted OR = 1.14, 95% CI 0.95–1.36, p = 0.157), nor between IST discontinuation and the type of infection (Table S5).

**Table 5:** Infections in ICU

Types of infections	Total (n = 124)
Bacterial infections at admission, n (%)	
Pneumonia	19 (15.3)
Transplant pyelonephritis	21 (16.9)
Colitis	3 (2.4)
Disseminated tuberculosis	1 (0.8)
Meningitis	1 (0.8)
Necrotizing fasciitis	1 (0.8)
Septic shock of undetermined origin	5 (4)
Angiocholitis	2 (1.6)
Viral infections at admission, n (%)	
SARS-CoV-2 pneumonia	44 (35.5)
Disseminated CMV infection	2 (1.6)
RSV pneumonia	1 (0.8)
VZV encephalitis	1 (0.8)
Fungal infection at admission, n (%)	
Cryptococcal meningitis	1 (0.8)
Pneumocystosis pneumonia	2 (1.6)
Parasitic infection at admission, n (%)	
Cerebral toxoplasmosis	1 (0.8)
Malaria	1 (0.8)
Concomitant infections at admission, n (%)	18 (14.4)
ICU acquired infections, n (%)	
Bacterial VAP	14 (11.3)
Transplant pyelonephritis	2 (1.6)
Aspergillosis pneumonia	4 (3.2)
Mucormycosis pneumonia	1 (0.8)
Aspergillosis sinusitis	1 (0.8)
Candidemia	1 (0.8)

CMV: Cytomegalovirus. RSV: Respiratory syncitial virus. VZV: Varicella zoster virus. VAP: Ventilation acquired pneumonia

Since SARS-CoV-2-related pneumonias have a different sepsis pathophysiology compared to bacterial infections, we conducted a subgroup analysis within this population. The discontinuation of at least one IST or CNI showed no statistically significant association with ICU mortality, AKI, MAKE at ICU discharge, or MAKE 180 (Table 6). Regarding MPA discontinuation, statistical analysis was not feasible because current guidelines recommend discontinuing MPA in severe cases of COVID-19 pneumonia. Consequently, no patients in this subgroup continued MPA therapy. To better understand the potential confounding effect of COVID-19–specific treatment practices, we extended our multivariable model to include SARS-CoV-2 pneumonia status and its interaction with MPA discontinuation. While the association between MPA discontinuation and the MAKE-180 outcome became borderline non-significant in this extended model (p = 0.056, Table S6), the absence of a statistically significant interaction (p = 0.61) suggests that the effect of MPA discontinuation did not differ substantially between COVID-19 and non–COVID-19 patients.

**Table 6:** Subgroup analysis of patients presenting SARS-CoV2 pneumonia for non-adjusted odds ratio of IST management on ICU mortality, AKI, MAKE at ICU discharge and MAKE 180

	ICU mortality		AKI		MAKE at ICU discharge		MAKE 180	
	OR (95% CI)	p	OR (95% CI)	p	OR (95% CI)	p	OR (95% CI)	p
At least 1 immunosuppressive drug discontinuation	1.117 (0.756–1.651)	0.582	1.113 (0.834–1.485)	0.471	1.044 (0.705–1.545)	0.831	1.137 (0.776–1.667)	0.514
CNI discontinuation	0.648 (0.438–0.958)	0.039	1.171 (0.901–1.523)	0.25	0.696 (0.472–1.028)	0.081	0.749 (0.514–1.09)	0.143
MPA discontinuation	NA	NA	NA	NA	NA	NA	NA	NA

IST: Immunosuppressive therapy; ICU: Intensive care unit; AKI: Acute kidney injury; CNI: Calcineurin inhibitor; MPA: Mycophenolic acid; NA: Non applicable

Additionally, we conducted a subgroup analysis of patients presenting with septic shock within 24 h of ICU admission, as defined by the Sepsis-3 criteria [27]. In univariate analysis, only MPA discontinuation was significantly associated with MAKE 180 (non-adjusted OR = 2.284, 95% CI 1.051–4.963, p = 0.049) (Table 7).

**Table 7:** Subgroup analysis of patients presenting a septic shock at admission for non-adjusted odds ratio of IST management on ICU mortality, AKI, MAKE at ICU discharge and MAKE 180

	ICU mortality		AKI		MAKE at ICU discharge		MAKE 180	
	OR (95% CI)	p	OR (95% CI)	p	OR (95% CI)	p	OR (95% CI)	p
At least 1 immunosuppressive drug discontinuation	1.084 (0.695–1.691)	0.724	1.108 (0.869–1.412)	0.416	0.979 (0.637–1.503)	0.922	1.227 (0.887–1.697)	0.225
CNI discontinuation	1.056 (0.741–1.507)	0.764	1.221 (1.004–1.486)	0.055	0.981 (0.698–1.378)	0.911	1.231 (0.959–1.58)	0.113
MPA discontinuation	1.76 (0.638–4.855)	0.287	0.917 (0.515–1.632)	0.77	1.92 (0.724–5.089)	0.203	2.284 (1.051–4.963)	0.049

IST: Immunosuppressive therapy; ICU: Intensive care unit; AKI: Acute kidney injury; CNI: Calcineurin inhibitor; MPA: Mycophenolic acid

## Discussion

This study is the first prospective multicenter study on the management of IST in KTRs hospitalized in ICU for sepsis. Our findings indicate that MPA discontinuation is significantly associated with MAKE 180, even after adjusting for patient severity and graft function at admission. We did not observe any significant association between IST continuation and ICU-acquired infections, nor between IST discontinuation and allograft rejection.

In our cohort, ICU mortality rate was 34.7%, aligning with previously reported retrospective studies [24, 37, 38]. The 1-year mortality rate in our cohort was 52.4%, despite a relatively low median SOFA score of 5 at admission. Comparative analysis of comorbidities revealed a higher prevalence of diabetes mellitus (53.2%) among our patients compared to other published cohorts [22, 23, 25]. To date, the 1-year mortality rate among KTRs admitted to the ICU for sepsis has not been thoroughly characterized in the literature. Bige et al. [23] previously reported a 3-month mortality rate of 22% in this population. We hypothesize that the elevated 1-year mortality observed in our cohort may be attributable to the high proportion of patients admitted with COVID-19-related complications. During the study period, ICU mortality among the general COVID-19 population in participating centers, ranged from 15 to 33%, whereas it reached 48% among kidney transplant recipients, highlighting the increased susceptibility of this population to severe outcomes. The requirement for organ support was similar in comparison to other studies, except for RRT which was less frequent in our cohort [23–25]. Additionally, the discontinuation of at least one immunosuppressive drug occurred more frequently in our cohort (80.6% of patients), with MPA being the most discontinued drug in 59.7% of cases. This contrasts with the discontinuation rates of 28% to 54.5% reported in other series [23–25]. Criteria for IST discontinuation were not predefined and instead reflected standard practices at individual centers, resulting in treatment heterogeneity. Discussions with investigators indicated that CNI discontinuation was mainly prompted by AKI or overdose, while MPA discontinuation was largely driven by neutropenia or concerns regarding excessive immunosuppression. The median time to CNI reinitiation was 4.5 days, exceeding the typical 24–48 h expected for isolated overdose, suggesting a deliberate reduction in immunosuppression. Even when overdose was cited as the reason, discontinuation often reflected a conscious clinical decision in the context of infection and organ dysfunction rather than a simple pharmacological adjustment. Nonetheless, the heterogeneity of CNI discontinuation indications limits interpretability, and conclusions regarding CNI management should be drawn with caution. Current guidelines suggest a benefit in reducing or stopping IST in solid organ transplant recipients in the context of sepsis [20, 30, 39]. However, these recommendations are based on limited retrospective data, and there is no consensus on which drug should be stopped, the timing and the duration of discontinuation. This lack of clear guidance explains the high heterogeneity of practices and the varying rates of drug discontinuation across studies [26]. Our study raises questions about these recommendations and supports previous retrospective data suggesting a detrimental effect of IST discontinuation in the ICU on patient survival and kidney function [23–25]. Specifically, we show that MPA discontinuation is associated with the composite outcome MAKE at 180 days. Although the association between MPA discontinuation and MAKE 180 cannot be definitively established, due to potential confounding factors that may have been overlooked, the prospective design of this study, the analysis of the timing of MPA discontinuation, and the adjustment for severity in the analysis allow us to hypothesize a potential detrimental effect of MPA discontinuation during sepsis. We acknowledge that despite performing a multivariable analysis, the treating physicians may have judged some patients as less severe and continued MPA, irrespective of the SOFA score. Indeed, among ICU patients, although there were no significant differences in terms of peak lactate values between those who continued MPA and those who discontinued it, patients who discontinued MPA required mechanical ventilation more frequently, and had longer ICU stays. It is important to emphasize that our observational study can only demonstrate statistical associations and cannot establish causal relationships. Moreover, this study was not specifically designed to assess the short-term clinical impact of IST discontinuation.

The immune response during sepsis involves an initial inflammatory phase that causes organ failures, followed by a prolonged immunosuppressive phase in severe cases, which promotes opportunistic infections even in non-immunocompromised patients. An alternative theory suggests that both inflammatory and immunosuppressive responses occur simultaneously, with the inflammatory response resolving earlier than the immunosuppressive phase [40, 41]. The use of drug-induced immunosuppression in solid organ transplant patients adds further complexity. One hypothesis to explain the association between MPA discontinuation and MAKE 180 outcome, is that reducing immunosuppression during the inflammatory phase of sepsis may result in immune system disinhibition, exacerbating this systemic inflammatory response. And it could result in more severe organ failures and, consequently, higher mortality. Nevertheless, we found an association between MPA discontinuation and ICU mortality, but not with RRT or persistent graft dysfunction. No association was found between MPA discontinuation and MAKE 360, which may reflect an outcome evaluated too long after ICU admission to adequately capture the impact of IST management. Additionally, the smaller number of survivors at 1 year may have limited the statistical power to identify differences at this endpoint.

Regarding CNIs, the association between CNI discontinuation and MAKE 180 lost statistical significance after adjusting for severity and kidney function at admission. This indicates that CNI discontinuation mainly occurred among the most severely ill patients with AKI at admission. In most cases, CNI discontinuation occurred within 24 h of ICU admission, coinciding with AKI diagnosis. This timing suggests that physicians might discontinue CNIs because of AKI, as CNIs are known to potentially exacerbate kidney injury [8, 42, 43].

In our cohort, 80.6% of patients experienced discontinuation of at least one immunosuppressive drug. Among survivors, only two cases of graft rejection occurred within the year following ICU admission, aligning with previously published data indicating the absence of association between IST discontinuation during sepsis and subsequent rejection [21, 22]. The prolonged immunosuppression induced by sepsis, which can persist several months after the initial infection [41] may account for low incidence of rejection following IST discontinuation in both our cohort and the existing literature. Indeed, dendritic cells loss and dysfunction [44–48], CD4+ T cells apoptosis and exhaustion [49–51], and B cell apoptosis [52, 53] have been described following sepsis. This suggests that sepsis could attenuate the development of an alloreactive response, thereby reducing the risk of graft rejection, even after temporary discontinuation of IST. Finally, the complex pathophysiology of sepsis, involving dynamic changes in immune response, underscores the current lack of effective tools to monitor patient immune status and guide physicians regarding the optimal timing and duration of drug discontinuation during and after sepsis.

Our study has several limitations that warrant consideration. First, the limited sample size reduces the statistical power of our findings, particularly when assessing the impact of IST discontinuation on clinical outcomes. Expanding the cohort would enhance the robustness and generalizability of our results. Second, part of the study coincided with SARS-CoV-2 pandemic, introducing confounding factors. Guidelines recommending the discontinuation of MPA in severe COVID-19 cases, particularly in patients also receiving dexamethasone and/or tocilizumab, influenced IST management. Although a subgroup analysis of COVID-19 cases found no association between IST discontinuation and MAKE 180, statistical analysis of MPA discontinuation was not possible due to its systematic suspension. Additionally, specific COVID-19 management data, such as vaccination status, use of monoclonal antibodies, or convalescent plasma, were not collected, limiting our analysis. Third, our study did not account for all aspects of patient management, such as increased corticosteroid doses potentially exacerbating immunosuppression. Moreover, to simplify our analysis, patients with IST dose reductions were included into the “IST continuation” group, as defining a clinically significant reduction threshold would have introduced considerable analytical complexity. Eighty KTRs were not included in the study, and since no outcome data were collected for them, we cannot assess whether their exclusion introduced selection bias. Furthermore, the lack of standardized IST protocols across centers resulted in IST management heterogeneity. This variability limits the comparability of patient subgroups and implies that our results describe associations rather than infer causal effects. We analyzed all sepsis together, regardless of whether the causative pathogen was bacterial, viral (including COVID-19), parasitic, or fungal. It is likely that IST management may affect the course of sepsis differently depending on the pathogen, but reaching this level of granularity would require larger, more powered studies. Finally, the lack of randomization inherent to the observational design introduces potential severity bias, as reflected by the heterogeneity in patient severity according to CNI and MPA discontinuation status.

## Conclusion

This study is the first multicentric prospective study suggesting an association between MPA discontinuation and adverse outcomes during sepsis in critically-ill KTRs. While these findings are hypothesis-generating, they must be interpreted with caution given the potential confounding introduced by SARS-Cov-2–specific treatment protocols. Our study is underpowered to draw firm conclusions in non-COVID-19 patients. These findings open new avenues for interventional studies, potentially incorporating immune response monitoring, to refine optimal IST management in this challenging clinical context.

## Supplementary Information

Supplementary material 1.

## Data Availability

The datasets used and/or analyzed during the current study are available from the corresponding author on reasonable request.

## Abbreviations

ADPKD: Autosomal Dominant Polycystic Kidney Disease
AKI: Acute kidney injury
AMR: Antibody mediated rejection
AZA: Azathioprine
C0: Residual cyclosporine level
CGN: Chronic glomerulonephritis
CNI: Calcineurin inhibitor
DSA: Donor specific antibody
eGFR: Estimated glomerular filtration rate
ESRD: End stage renal disease
ICU: Intensive Care Unit
mTORi: Inhibitor of mammalian Target Of Rapamycin
IQR: Interquartile range
IST: Immunosuppressive treatment
KTR: Kidney transplant recipient
MAKE: Major adverse kidney event
MPA: Mycophenolic acid
OI: Opportunistic infection
OR: Odds ratio
RRT: Renal replacement therapy
SOFA: Sepsis-related Organ Failure Assessment
T0: Residual tacrolimus level
TCMR: T cell mediated rejection
VAP: Ventilation acquired pneumonia

## References

[1] Laupacis A, Keown P, Pus N, Krueger H, Ferguson B, Wong C, et al. A study of the quality of life and cost-utility of renal transplantation. Kidney Int. 1996;50:235–42. 8807593 10.1038/ki.1996.307

[2] Coemans M, Süsal C, Döhler B, Anglicheau D, Giral M, Bestard O, et al. Analyses of the short- and long-term graft survival after kidney transplantation in Europe between 1986 and 2015. Kidney Int. 2018;94:964–73. 30049474 10.1016/j.kint.2018.05.018

[3] Oniscu GC, Brown H, Forsythe JLR. Impact of cadaveric renal transplantation on survival in patients listed for transplantation. J Am Soc Nephrol. 2005;16:1859–65. 15857921 10.1681/ASN.2004121092

[4] Abecassis M, Bartlett ST, Collins AJ, Davis CL, Delmonico FL, Friedewald JJ, et al. Kidney transplantation as primary therapy for end-stage renal disease: a National Kidney Foundation/Kidney Disease Outcomes Quality Initiative (NKF/KDOQITM) conference. Clin J Am Soc Nephrol. 2008;3:471–80. 18256371 10.2215/CJN.05021107 PMC2390948

[5] Port FK, Wolfe RA, Mauger EA, Berling DP, Jiang K. Comparison of survival probabilities for dialysis patients vs cadaveric renal transplant recipients. JAMA. 1993;270:1339–43. 8360969

[6] Wolfe RA, Ashby VB, Milford EL, Ojo AO, Ettenger RE, Agodoa LY, et al. Comparison of mortality in all patients on dialysis, patients on dialysis awaiting transplantation, and recipients of a first cadaveric transplant. N Engl J Med. 1999;341:1725–30. 10580071 10.1056/NEJM199912023412303

[7] Canet E, Osman D, Lambert J, Guitton C, Heng A-E, Argaud L, et al. Acute respiratory failure in kidney transplant recipients: a multicenter study. Crit Care. 2011;15:R91. 21385434 10.1186/cc10091 PMC3219351

[8] Graham RM. Cyclosporine: mechanisms of action and toxicity. Cleve Clin J Med. 1994;61:308–13. 7923750 10.3949/ccjm.61.4.308

[9] Thomson AW, Bonham CA, Zeevi A. Mode of action of tacrolimus (FK506): molecular and cellular mechanisms. Ther Drug Monit. 1995;17:584–91. 8588225 10.1097/00007691-199512000-00007

[10] Vincenti F, Larsen C, Durrbach A, Wekerle T, Nashan B, Blancho G, et al. Costimulation blockade with belatacept in renal transplantation. N Engl J Med. 2005;353:770–81. 16120857 10.1056/NEJMoa050085

[11] Larsen CP, Pearson TC, Adams AB, Tso P, Shirasugi N, Strobert E, et al. Rational development of LEA29Y (belatacept), a high-affinity variant of CTLA4-Ig with potent immunosuppressive properties. Am J Transplant. 2005;5:443–53. 15707398 10.1111/j.1600-6143.2005.00749.x

[12] Allison AC. Mechanisms of action of mycophenolate mofetil. Lupus. 2005;14(Suppl 1):s2-8. 15803924 10.1191/0961203305lu2109oa

[13] Chan GLC, Canafax DM, Johnson CA. The therapeutic use of azathioprine in renal transplantation. Pharmacotherapy. 1987;7:165–77. 3324057 10.1002/j.1875-9114.1987.tb04046.x

[14] Sehgal SN. Sirolimus: its discovery, biological properties, and mechanism of action. Transpl Proc. 2003;35:S7-14. 10.1016/s0041-1345(03)00211-2 12742462

[15] Moes DJAR, Guchelaar H-J, de Fijter JW. Sirolimus and everolimus in kidney transplantation. Drug Discov Today. 2015;20:1243–9. 26050578 10.1016/j.drudis.2015.05.006

[16] Cain DW, Cidlowski JA. Immune regulation by glucocorticoids. Nat Rev Immunol. 2017;17:233–47. 28192415 10.1038/nri.2017.1 PMC9761406

[17] El-Zoghby ZM, Stegall MD, Lager DJ, Kremers WK, Amer H, Gloor JM, et al. Identifying specific causes of kidney allograft loss. Am J Transplant. 2009;9:527–35. 19191769 10.1111/j.1600-6143.2008.02519.x

[18] Kahwaji J, Bunnapradist S, Hsu J-W, Idroos ML, Dudek R. Cause of death with graft function among renal transplant recipients in an integrated healthcare system. Transplantation. 2011;91:225–30. 21048529 10.1097/TP.0b013e3181ff8754

[19] Al-Hasan MN, Razonable RR, Kremers WK, Baddour LM. Impact of Gram-negative bloodstream infection on long-term allograft survival after kidney transplantation. Transplantation. 2011;91:1206–10. 21494179 10.1097/TP.0b013e3182180535

[20] Maggiore U, Abramowicz D, Crespo M, Mariat C, Mjoen G, Peruzzi L, et al. How should I manage immunosuppression in a kidney transplant patient with COVID-19? An ERA-EDTA DESCARTES expert opinion. Nephrol Dial Transplant. 2020;35:899–904. 32441741 10.1093/ndt/gfaa130 PMC7313836

[21] Sun Q, Liu Z-H, Chen J, Ji S, Tang Z, Cheng Z, et al. An aggressive systematic strategy for acute respiratory distress syndrome caused by severe pneumonia after renal transplantation. Transpl Int. 2006;19:110–6. 16441359 10.1111/j.1432-2277.2005.00245.x

[22] Shih C-J, Tarng D-C, Yang W-C, Yang C-Y. Immunosuppressant dose reduction and long-term rejection risk in renal transplant recipients with severe bacterial pneumonia. Singapore Med J. 2014;55:372–7. 25091886 10.11622/smedj.2014089 PMC4291963

[23] Bige N, Zafrani L, Lambert J, Peraldi M-N, Snanoudj R, Reuter D, et al. Severe infections requiring intensive care unit admission in kidney transplant recipients: impact on graft outcome. Transpl Infect Dis. 2014;16:588–96. 24966154 10.1111/tid.12249

[24] Kim HD, Chung BH, Yang CW, Kim SC, Kim KH, Kim SY, et al. Management of immunosuppressive therapy in kidney transplant recipients with sepsis: a multicenter retrospective study. J Intensive Care Med. 2024;39(8):758–67. 38321761 10.1177/08850666241231495

[25] Guinault D, Del Bello A, Lavayssiere L, Nogier M-B, Cointault O, Congy N, et al. Outcomes of kidney transplant recipients admitted to the intensive care unit: a retrospective study of 200 patients. BMC Anesthesiol. 2019;19:130. 31315561 10.1186/s12871-019-0800-0 PMC6637509

[26] Shepshelovich D, Tau N, Green H, Rozen-Zvi B, Issaschar A, Falcone M, et al. Immunosuppression reduction in liver and kidney transplant recipients with suspected bacterial infection: a multinational survey. Transpl Infect Dis. 2019;21: e13134. 31242341 10.1111/tid.13134

[27] Singer M, Deutschman CS, Seymour CW, Shankar-Hari M, Annane D, Bauer M, et al. The Third International Consensus Definitions for sepsis and septic shock (Sepsis-3). JAMA. 2016;315:801–10. 26903338 10.1001/jama.2016.0287 PMC4968574

[28] Vincent JL, Moreno R, Takala J, Willatts S, De Mendonça A, Bruining H, et al. The SOFA (Sepsis-related Organ Failure Assessment) score to describe organ dysfunction/failure. On behalf of the Working Group on Sepsis-Related Problems of the European Society of Intensive Care Medicine. Intensive Care Med. 1996;22:707–10. 8844239 10.1007/BF01709751

[29] Levey AS, Stevens LA, Schmid CH, Zhang YL, Castro AF, Feldman HI, et al. A new equation to estimate glomerular filtration rate. Ann Intern Med. 2009;150:604–12. 19414839 10.7326/0003-4819-150-9-200905050-00006 PMC2763564

[30] Kasiske BL, Zeier MG, Chapman JR, Craig JC, Ekberg H, Garvey CA, et al. KDIGO clinical practice guideline for the care of kidney transplant recipients: a summary. Kidney Int. 2010;77:299–311. 19847156 10.1038/ki.2009.377

[31] Loupy A, Haas M, Roufosse C, Naesens M, Adam B, Afrouzian M, et al. The Banff 2019 Kidney Meeting Report (I): updates on and clarification of criteria for T cell- and antibody-mediated rejection. Am J Transplant. 2020;20:2318–31. 32463180 10.1111/ajt.15898 PMC7496245

[32] European Centre for Disease Prevention and Control. Surveillance of healthcare-associated infections in intensive care units: HAI Net ICU protocol, version 2.2. LU: Publications Office; 2017. 10.2900/833186

[33] Billings FT, Shaw AD. Clinical trial endpoints in acute kidney injury. Nephron Clin Pract. 2014;127:89–93. 25343828 10.1159/000363725 PMC4480222

[34] Pickkers P, Angus DC, Bass K, Bellomo R, van den Berg E, Bernholz J, et al. Phase-3 trial of recombinant human alkaline phosphatase for patients with sepsis-associated acute kidney injury (REVIVAL). Intensive Care Med. 2024;50:68–78. 38172296 10.1007/s00134-023-07271-w PMC10810941

[35] Vinclair C, De Montmollin E, Sonneville R, Reuter J, Lebut J, Cally R, et al. Factors associated with major adverse kidney events in patients who underwent veno-arterial extracorporeal membrane oxygenation. Ann Intensive Care. 2020;10:44. 32307616 10.1186/s13613-020-00656-w PMC7167383

[36] Bhatraju PK, Zelnick LR, Chinchilli VM, Moledina DG, Coca SG, Parikh CR, et al. Association between early recovery of kidney function after acute kidney injury and long-term clinical outcomes. JAMA Netw Open. 2020;3: e202682. 32282046 10.1001/jamanetworkopen.2020.2682 PMC7154800

[37] Mouloudi E, Massa E, Georgiadou E, Iosifidis E, Kydona C, Sgourou K, et al. Course and outcome of renal transplant recipients admitted to the intensive care unit: a 20-year study. Transpl Proc. 2012;44:2718–20. 10.1016/j.transproceed.2012.09.097 23146503

[38] Arulkumaran N, West S, Chan K, Templeton M, Taube D, Brett SJ. Long-term renal function and survival of renal transplant recipients admitted to the intensive care unit. Clin Transplant. 2012;26:E24-31. 21955177 10.1111/j.1399-0012.2011.01520.x

[39] Kalil AC, Dakroub H, Freifeld AG. Sepsis and solid organ transplantation. Curr Drug Targets. 2007;8:533–41. 17430124 10.2174/138945007780362746

[40] van der Poll T, van de Veerdonk FL, Scicluna BP, Netea MG. The immunopathology of sepsis and potential therapeutic targets. Nat Rev Immunol. 2017;17:407–20. 28436424 10.1038/nri.2017.36

[41] Venet F, Monneret G. Advances in the understanding and treatment of sepsis-induced immunosuppression. Nat Rev Nephrol. 2018;14:121–37. 29225343 10.1038/nrneph.2017.165

[42] de Mattos AM, Olyaei AJ, Bennett WM. Nephrotoxicity of immunosuppressive drugs: long-term consequences and challenges for the future. Am J Kidney Dis. 2000;35:333–46. 10676738 10.1016/s0272-6386(00)70348-9

[43] Naesens M, Kuypers DRJ, Sarwal M. Calcineurin inhibitor nephrotoxicity. Clin J Am Soc Nephrol. 2009;4:481–508. 19218475 10.2215/CJN.04800908

[44] Grimaldi D, Louis S, Pène F, Sirgo G, Rousseau C, Claessens YE, et al. Profound and persistent decrease of circulating dendritic cells is associated with ICU-acquired infection in patients with septic shock. Intensive Care Med. 2011;37:1438–46. 21805160 10.1007/s00134-011-2306-1

[45] Kumar V. Dendritic cells in sepsis: potential immunoregulatory cells with therapeutic potential. Mol Immunol. 2018;101:615–26. 30007546 10.1016/j.molimm.2018.07.007

[46] Guisset O, Dilhuydy M-S, Thiébaut R, Lefèvre J, Camou F, Sarrat A, et al. Decrease in circulating dendritic cells predicts fatal outcome in septic shock. Intensive Care Med. 2007;33:148–52. 17091240 10.1007/s00134-006-0436-7

[47] Fan X, Liu Z, Jin H, Yan J, Liang H. Alterations of dendritic cells in sepsis: featured role in immunoparalysis. Biomed Res Int. 2015;2015: 903720. 25821827 10.1155/2015/903720 PMC4363672

[48] Faivre V, Lukaszewicz AC, Alves A, Charron D, Payen D, Haziot A. Human monocytes differentiate into dendritic cells subsets that induce anergic and regulatory T cells in sepsis. PLoS ONE. 2012;7: e47209. 23071758 10.1371/journal.pone.0047209 PMC3468528

[49] Inoue S, Suzuki-Utsunomiya K, Okada Y, Taira T, Iida Y, Miura N, et al. Reduction of immunocompetent T cells followed by prolonged lymphopenia in severe sepsis in the elderly. Crit Care Med. 2013;41:810–9. 23328259 10.1097/CCM.0b013e318274645f

[50] Heffernan DS, Monaghan SF, Thakkar RK, Machan JT, Cioffi WG, Ayala A. Failure to normalize lymphopenia following trauma is associated with increased mortality, independent of the leukocytosis pattern. Crit Care. 2012;16:R12. 22264310 10.1186/cc11157 PMC3396248

[51] Ono S, Tsujimoto H, Hiraki S, Aosasa S. Mechanisms of sepsis-induced immunosuppression and immunological modification therapies for sepsis. Ann Gastroenterol Surg. 2018;2:351–8. 30238076 10.1002/ags3.12194 PMC6139715

[52] Shankar-Hari M, Fear D, Lavender P, Mare T, Beale R, Swanson C, et al. Activation-associated accelerated apoptosis of memory B cells in critically ill patients with sepsis. Crit Care Med. 2017;45:875–82. 28296810 10.1097/CCM.0000000000002380

[53] Hotchkiss RS, Monneret G, Payen D. Sepsis-induced immunosuppression: from cellular dysfunctions to immunotherapy. Nat Rev Immunol. 2013;13:862–74. 24232462 10.1038/nri3552 PMC4077177

