# Supplementary material for: Immunosuppressive therapy management during sepsis in kidney transplant recipients: a prospective multicenter study
Source: Ann Intensive Care. 2025 Aug 10;15:116. doi: 10.1186/s13613-025-01523-2 (PMC12336102; doi:10.1186/s13613-025-01523-2)
Supplement: Supplementary file 1 — Supplementary material 1. [file 13613_2025_1523_MOESM1_ESM.docx]

**SUPPLEMENTAL TABLES**

**Table S1: Patients characteristics by MAKE 180 outcome components (death, persistent graft dysfunction, need for renal replacement**

|  | MAKE 180 = 1  (n=85) | MAKE 180 =  Death  (n=62) | MAKE 180 =  Graft dysfunction  (n=16) | MAKE 180 =  Need for RRT  (n=7) |
| --- | --- | --- | --- | --- |
| Median age (IQR), years | 65 (57-71) | 66 (57-71) | 53.5 (40.75-65) | 49 (43-66) |
| Gender, male, n (%) | 42 (49.4) | 35 (56.4) | 8 (50) | 3 (42.9) |
| ESRD etiology, n (%)  Diabetes mellitus  Hypertension  CGN  ADPKD  Others | 26 (30.6)  9 (10.6)  18 (21.2)  4 (4.7)  28 (32.9) | 18 (29)  7 (11.3)  13 (20.9)  4 (6.5)  20 (32.3) | 5 (31.3)  2 (12.5)  2 (12.5)  0 (0)  7 (43.8) | 3 (42.9)  0 (0)  3 (42.9)  0 (0)  1 (14.3) |
| Modality of pre-transplant dialysis, n (%)  Hemodialysis  Peritoneal dialysis | 85 (100)  0 (0) | 62 (100)  0 (0) | 16 (100)  0 (0) | 7 (100)  0 (0) |
| Comorbidities, n (%)  Malignancy  Other cause of immunosuppression  Coronary artery disease  Cerebrovascular disease  Peripheral artery disease  Diabetes mellitus  Hypertension | 19 (22.4)  11 (12.9)  19 (22.4)  5 (5.9)  15 (17.7)  49 (57.7)  74 (87.7) | 15 (24.2)  9 (14.5)  15 (24.2)  5 (8)  7 (11.3)  34 (54.8)  54 (87.1) | 5 (31.3)  5 (31.3)  1 (6.3)  0 (0)  2 (12.5)  7 (43.8)  16 (100) | 2 (28.6)  2 (28.6)  1 (14.3)  0 (0)  1 (14.3)  4 (57.1)  7 (100) |
| History of allograft rejection, n (%)  TCMR  AMR | 7 (8.2)  14 (16.5) | 3 (4.8)  10 (12.1) | 2 (12.5)  4 (25) | 2 (28.6)  0 (0) |
| History of DSA (n= 62)  DSA  No DSA | 11 (31.4)  24 (68.6) | 7 (11.3)  16 (25.8) | 2 (12.5)  6 (37.5) | 2 (28.6)  2 (28.6) |
| History of opportunistic infection, n (%)  Viral OI  Bacterial OI  Fungal OI | 42 (49.4)  22 (25.9)  2 (2.4)  9 (10.6) | 34 (54.8)  18 (29)  0 (0)  6 (9.7) | 6 (37.5)  3 (18.8)  1 (6.3)  2 (12.5) | 2 (28.6)  1 (14.3)  1 (14.3)  1 (14.3) |
| Median time between transplantation and ICU admission (IQR), months | 71.5 (27-121) | 55 (21.5-115.5) | 74 (55-136) | 74 (62-119) |
| Median creatinine at baseline (IQR), µmol/L  Median eGFR at baseline (IQR), ml/min/1.73 m^2^ | 147 (107-200)  39 (27.6-56.9) | 149 (110-200)  38 (27.45-58.48) | 116.5 (97.8-213.3)  58 (26-68.7) | 208 (100-222)  31.8 (19.5-69.2) |
| Baseline immunosuppressive regimen, n (%)  CNI  MPA  AZA  mTORi  Corticosteroids  Belatacept | 72 (84.7)  59 (69.4)  6 (7.1)  9 (10.6)  74 (87.1)  8 (9.4) | 52 (83.9)  42 (67.7)  4 (6.5)  7 (11.3)  53 (85.5)  6 (9.7) | 14 (87.5)  11 (68.8)  2 (12.5)  2 (12.5)  14 (87.5)  1 (6.3) | 6 (85.7)  6 (85.7)  0 (0)  0 (0)  7 (0)  1 (14.3) |
| Number of immunosuppressive drugs at admission, n (%)  4  3  2  1 | 1 (1.2)  57 (67.1)  26 (30.6)  1 (1.2) | 0 (0)  40 (64.5)  22 (35.5)  0 (0) | 1 (6.3)  11 (68.8)  3 (18.8)  1 (6.3) | 0 (0)  6 (85.7)  1 (14.3)  0 (0) |
| Median duration of ICU stay (IQR), days | 8.4 (4-15) | 9.5 (4-15) | 7.5 (4-21.5) | 7.5 (5.5-9.3) |
| Median SOFA score at ICU admission (IQR) | 6 (5-8) | 5.5 (5-9) | 6 (3.25-9.75) | 6 (5-7) |
| Cause of ICU admission, n (%)  Hemodynamic failure  Respiratory failure  Neurological failure | 27 (31.8)  55 (64.7)  3 (3.5) | 19 (30.6)  42 (67.7)  1 (1.6) | 5 (31.3)  10 (12.1)  1 (6.3) | 3 (42.9)  3 (42.9)  1 (14.3) |
| IST at ICU admission, n (%)  CNI  MPA  AZA  mTORi  Corticosteroids | 70 (82.4)  43 (50.6)  4 (4.7)  10 (11.8)  76 (89.4) | 51 (82.3)  29 (46.8)  2 (3.2)  7 (11.3)  56 (90.3) | 14 (87.5)  10 (62.5)  2 (12.5)  2 (12.5)  15 (93.8) | 5 (71.4)  4 (57.1)  0 (0)  1 (14.3)  5 (71.4 |
| Median IST dosage at ICU admission (IQR)  Tacrolimus T0, ng/mL (n=66)  Ciclosporin C0, ng/mL (n=15)  MPA, mg per day (n=62)  Prednisone, mg per day (n=81) | 6.8 (5-9.9)  78 (55.5-132.5)  1000 (1000-1500)  7.5 (5-10) | 6.7 (4.9-9.6)  78 (52-109)  1000 (1000-1500)  10 (5-10) | 9.1 (3.9-21)  66 (25-344)  1000 (875-1500)  7.5 (5-10) | 6.6 (4.86-14.3)  156 (NA)  1000 (1000-2000)  5 (5-12.5) |

ESRD: End Stage Renal Disease. CGN: Chronic Glomerulonephritis. ADPKD: Autosomal Dominant Polycystic Kidney Disease. TCMR: T cell mediated rejection. AMR: Antibody Mediated Rejection. DSA: Donor specific Antibodies. OI: Opportunistic infection. eGFR: estimated Glomerular Filtration Rate. CNI: Calcineurin Inhibitor. MPA: Mycophenolic Acid. AZA: Azathioprine. mTORi: mTOR inhibitor. SOFA: Sepsis-related Organ Failure Assessment.

**Table S2: Details on MAKE outcomes according to IST management**

|  | MAKE at ICU discharge =1  (n=54) | MAKE at ICU discharge =0  (n=70) | MAKE 180 =1  (n=85) | MAKE 180 =0  (n=38) | MAKE 360 =1  (n=89) | MAKE 360 =0  (n=32) |
| --- | --- | --- | --- | --- | --- | --- |
| IST discontinuation, n (%)  IST continuation, n (%) | 47 (87)  7 (13) | 53 (76)  17 (24) | 75 (88)  10 (12) | 25 (66)  13 (34) | 74 (83)  15 (17) | 24 (75)  8 (25) |
| CNI discontinuation, n (%)  CNI continuation, n (%) | 20 (37)  26 (48) | 20 (26)  37 (53) | 33 (39)  39 (46) | 7 (18)  23 (61) | 32 (36)  43 (48) | 7 (22)  18 (56) |
| MPA discontinuation, n (%)  MPA continuation, n (%) | 35 (65)  0 (0) | 39 (56)  10 (14) | 52 (61)  2 (2) | 22 (56)  8 (21) | 51 (57)  5 (6) | 21 (66)  5 (16) |

IST: Immunosuppressive Therapy. CNI: Calcineurin Inhibitor. MPA: Mycophenolic Acid.

**Table S3: Non-adjusted Odds Ratio of minimal discontinuation time of IST, and number of discontinued IST on MAKE 180 criteria**

| IST management | MAKE 180 | |
| --- | --- | --- |
|  | OR (95% CI) | p |
| CNI discontinuation >24H  CNI discontinuation >48H  CNI discontinuation >72H | 1.21 (1.007-1.453) | 0.045 |
|  | 1.176 (0.737-1.875) | 0.501 |
|  | 1.273 (0.943-1.720) | 0.124 |
| MPA discontinuation >24H  MPA discontinuation >48H  MPA discontinuation >72H | 1.653 (1.22-2.234) | 0.001 |
|  | 1.432 (1.093-1.877) | 0.011 |
|  | 1.447 (1.143-1.832) | 0.003 |
| Reduction from 3 to 2 drugs  Reduction from 3 to 1 drug  Reduction from 2 to 1 drug | 0.973 (0.821-1.153) | 0.753 |
|  | 1.118 (0.919-1.362) | 0.273 |
|  | 1.154 (0.928-1.433) | 0.2 |

IST: Immunosuppressive Therapy. CNI: Calcineurin Inhibitor. MPA: Mycophenolic Acid.

**Table S4: Mean variation of eGFR at 180 and 360 days according to IST management**

|  | IST discontinuation | IST continuation | p | CNI discontinuation | CNI continuation | p | MPA discontinuation | MPA continuation | p |
| --- | --- | --- | --- | --- | --- | --- | --- | --- | --- |
| Mean ΔeGFR at 180 days, ml/min/1.73m^2^ | -6.65 | -3.94 | 0.43 | -16.94 | -3.18 | 0.013 | -2.68 | -6.02 | 0.4 |
| Mean ΔeGFR at 360 days, ml/min/1.73m^2^ | -8.84 | -8.57 | 0.95 | -16.09 | -7.2 | 0.12 | -5.99 | -10.47 | 0.34 |

IST: Immunosuppressive Therapy. CNI: Calcineurin Inhibitor. MPA: Mycophenolic Acid. ΔeGFR : Variation of estimated Glomerular Filtration Rate according to baseline value.

**Table S5: IST management according to the type of infection during ICU stay**

|  | IST discontinuation  (n=100) | IST continuation  (n=24) | p | CNI discontinuation  (n=40) | CNI continuation  (n=63) | p | MPA discontinuation  (n=74) | MPA continuation  (n=10) | p |
| --- | --- | --- | --- | --- | --- | --- | --- | --- | --- |
| Bacterial infection, n (%), (n=53) | 41 (41) | 12 (50) | 0.568 | 21 (53) | 26 (41) | 0.362 | 28 (38) | 9 (90) | 0.005 |
| Viral infection, n (%), (n=35) | 25 (25) | 10 (42) | 0.13 | 8 (20) | 18 (29) | 0.457 | 20 (27) | 1 (10) | 0.437 |
| Fungal infection, n (%), (n=2) | 2 (2) | 0 (0) | 1 | 0 (0) | 1 (2) | 1 | 2 (3) | 0 (0) | 1 |
| Parasitic infection, n (%), (n=1) | 0 (0) | 1 (4) | 0.194 | 0 (0) | 1 (2) | 1 | 0 (0) | 0 (0) | NA |
| Combined infections, n (%), (n=33) | 32 (32) | 1 (4) | 0,004 | 11 (28) | 17 (27) | 1 | 24 (32) | 0 (0) | 0.056 |

IST: Immunosuppressive Therapy. CNI: Calcineurin Inhibitor. MPA: Mycophenolic Acid.

**Table S6:** **Factors associated with MAKE 180 by multivariable analysis including SARS-CoV-2**

|  | OR (CI 95%) | p |
| --- | --- | --- |
| Extra renal SOFA (per point) | 1.039 (0.002 – 0.076) | 0.0399 |
| Age (per year) | 1.007 (1.000 – 1.014) | 0.0572 |
| Serum creatinine at admission (per µmol/L) | 1.000 (1.000 – 1.001) | 0.0391 |
| Male sex | 0.726 (0.596 – 0.884) | 0.0021 |
| MPA discontinuation | 1.339 (0.997 – 1.799) | 0.0565 |
| SARS-CoV-2 positivity | 1.240 (1.008 – 1.526) | 0.0450 |
| MPA discontinuation * SARS-CoV-2 positivity interaction | NA | 0.6134 |

MPA: Mycophenolic Acid.

**SUPPLEMENTAL FIGURES**

**
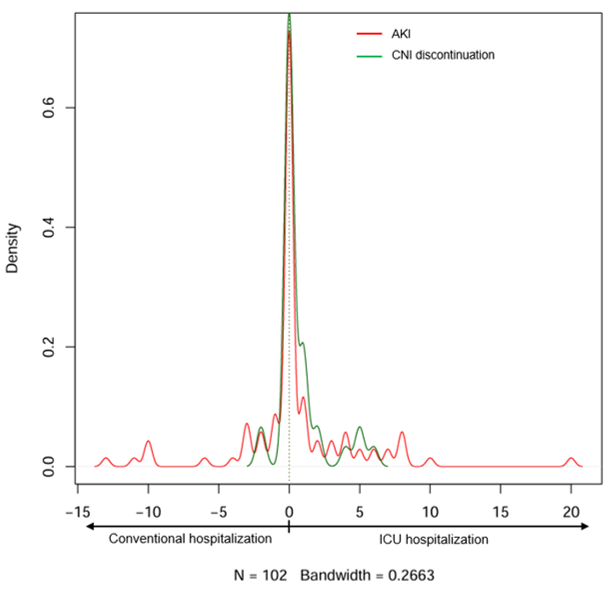
**

**Figure S1: Density curve of time between ICU admission and AKI occurrence and CNI discontinuation**

Time is expressed as days. Negative values represent days in conventional hospitalization, the value 0 represents the day of ICU admission, and positive values represent the days in ICU. Red line represents AKI occurrence, and green line represents CNI discontinuation occurrence. AKI : Acute Kidney Injury. CNI : Calcineurin inhibitor


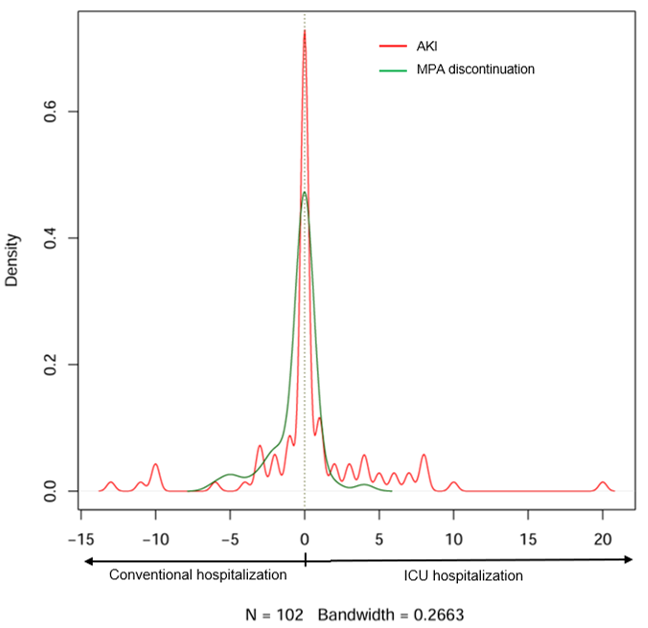


**Figure S2: Density curve of time between ICU admission and AKI and MPA discontinuation**

Time is expressed as days. Negative values represent days in conventional hospitalization, the value 0 represents the day of ICU admission, and positive values represent the days in ICU. Red line represents AKI occurrence, and green line represents MPA discontinuation occurrence. AKI : Acute Kidney Injury. MPA : Mycophenolic Acid


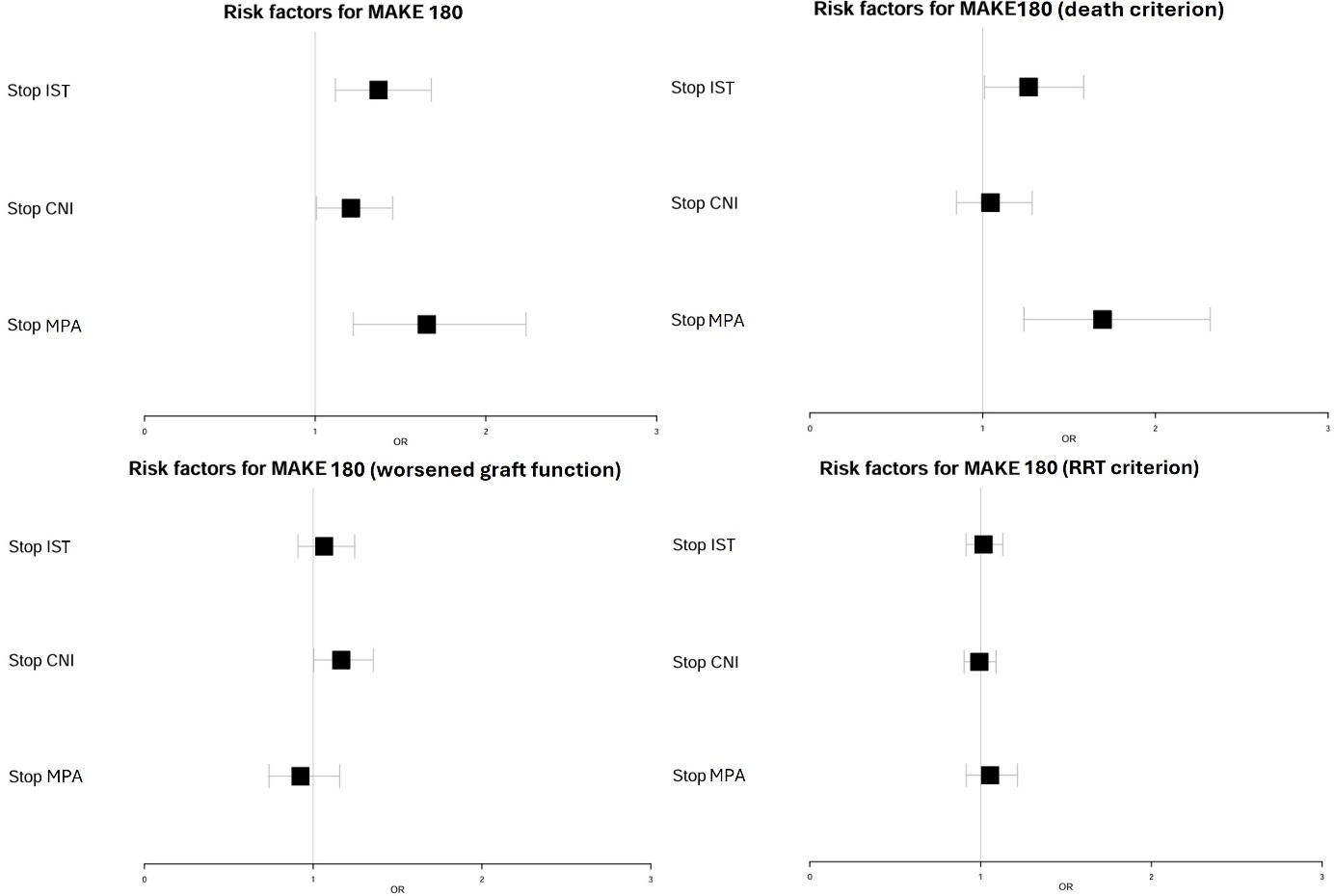


**Figure S3: Forest Plot of the individual components of MAKE 180 according to IST management**

IST: Immunosuppressive Therapy. CNI: Calcineurin Inhibitor. MPA: Mycophenolic Acid. RRT: Renal Replacement Therapy


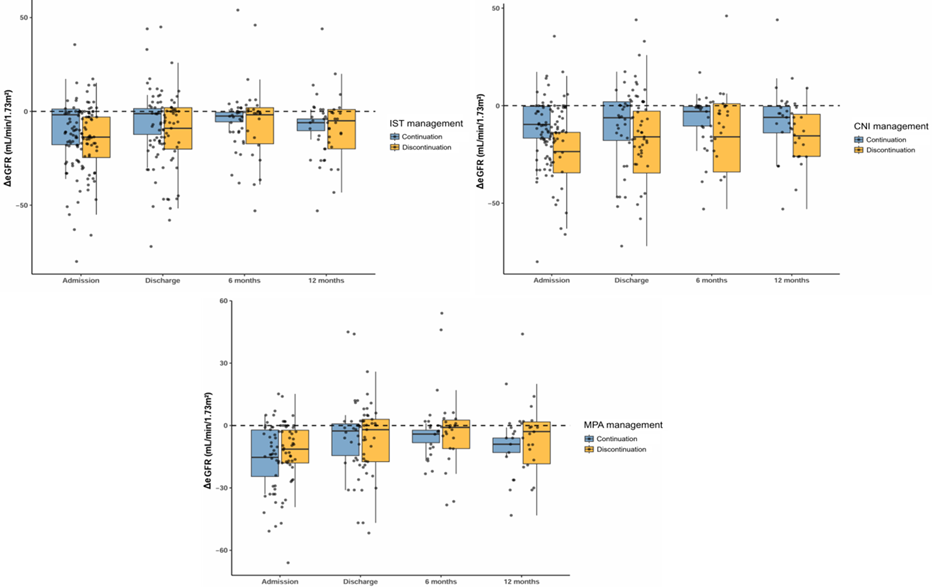


**Figure S4: Box plot of eGFR variations relative to baseline eGFR**

IST: Immunosuppressive Therapy. CNI: Calcineurin Inhibitor. MPA: Mycophenolic Acid. ΔeGFR : Variation of estimated Glomerular Filtration Rate relative to baseline value.
